# Toward dynamic Monte Carlo source modeling: measurement variability and primary electron source-width sensitivity in very small photon fields

**DOI:** 10.3389/fonc.2026.1968378

**Published:** 2026-10-28

**Authors:** Mina M. Habib

**Affiliations:** Department of Radiation Physics, Maadi Armed Forces Medical Compound, Cairo, Egypt

**Keywords:** angular divergence, BEAMnrc, dynamic source, EGSnrc, photon phase space, primary electron source, small-field dosimetry, source width

## Abstract

**Background:**

Very small photon fields are sensitive to both measurement conditions and radiation-source modelling, but the connection between effective primary-electron source width, downstream photon angular phase space, and field-size-dependent dosimetric response remains incompletely characterized.

**Methods:**

The study was restricted to 6 MV. Empirical measurement variability was evaluated using five ionization-chamber datasets acquired on a clinical Elekta Versa HD for fields from 20×20 to 3 × 3 cm^2^. A generalized Versa HD/Agility EGSnrc/BEAMnrc model was evaluated with two prescribed stationary Gaussian source endpoints, M1 (FWHM*_x_* = FWHM*_y_* = 0.15 cm) and M2 (FWHM*_x_* = 0.35 cm, FWHM*_y_* = 0.60 cm). DOSXYZnrc dose responses and final pre-phantom photon phase-space data were analysed for 3×3, 2×2, 1×1, and 0.5×0.5 cm^2^. The 3×3 cm^2^ field provided the experimental–Monte Carlo overlap; the smaller fields were controlled Monte Carlo endpoint-sensitivity analyses. M1 was additionally benchmarked against a 10×10 cm^2^ CC13 commissioning PDD.

**Results:**

Mean experimental coefficient of variation increased from 2.46% at 20×20 cm^2^ to 15.45% at 3×3 cm^2^. The M2 central-axis reductions were 8.48%, 8.27%, 29.59%, and 56.34% for 3×3, 2×2, 1×1, and 0.5×0.5 cm^2^, respectively. The corresponding M2/M1 ratios of the 95% angular-containment solid angle were 1.275, 1.498, 2.415, and 3.835. The four-field comparison demonstrates clear field-size progression in angular containment, while showing that amplification is observable-dependent rather than universally monotonic. The M1 PDD benchmark supported reference depth-dose behaviour, whereas lateral-profile findings were restricted to controlled numerical sensitivity because absolute lateral agreement was not independently established in this study alone.

**Conclusion:**

The demonstrated result is a stationary endpoint-sensitivity chain linking effective primary-electron source representation, linac-head transport, photon angular phase space, and small-field dose/profile response. A reduced covariance formulation is retained only as a prospective framework for testing whether a stationary effective source can represent unresolved accelerator-source states; no time-resolved source dynamics were measured or simulated.

## 1 Introduction

Small radiation fields are widely used in stereotactic radiotherapy, radiosurgery, and highly modulated treatments. They allow high dose conformity and steep dose gradients around the target. However, small-field dosimetry remains difficult because several physical and measurement effects become more important as the field size decreases. These include loss of lateral charged-particle equilibrium, steep dose gradients, detector volume averaging, detector perturbation, and field-size-dependent detector response. In addition, the accuracy of the calculated dose depends on the quality of beam modelling used in the treatment planning system or Monte Carlo model (1–4).

For this reason, disagreement in very small fields should not be considered only as a detector problem. Recent studies have shown that important calculation differences may remain even when small-field detectors show close agreement. Fogliata et al. reported close detector agreement while important model-dependent differences remained for the smallest fields in a ring-linac beam model (5). Nishioka et al. also showed that calculated small-field output factors depend on beam-modelling strategy and on the measurement information included during commissioning (3). These observations indicate that both measurement and beam-model representation contribute to the small-field problem.

One important part of beam modelling is the representation of the radiation source. Borzov et al. reconstructed an incident electron-source intensity distribution and showed that source representation affects calculated output factors when partial source occlusion becomes important (6). Their reconstructed source was described by an elliptical double-Gaussian distribution. More recently, Fogliata et al. showed that effective spot-size parameters used in Acuros influence small-field output factors, dosimetric field size, and penumbra (7). These studies support the importance of source representation in small-field dose calculation.

Our previous Monte Carlo study also showed that changing the primary electron source width can produce important changes in small-field dose profiles and penumbra (8). In that work, M2 was deliberately defined as a large asymmetric modification of the narrow M1 source—0.35 cm in *x* and 0.60 cm in *y*—to test the dosimetric consequences of a controlled departure from the idealized symmetric source, rather than to represent a measured clinical source distribution. The present study retains those same endpoint models and addresses a different question. Instead of examining only the final dose-profile response, it also investigates how that prescribed source-width perturbation appears in the photon angular phase space before the phantom.

Two widely used clinical dose-calculation systems illustrate the separation between source modelling and downstream particle transport. In Eclipse, Acuros XB starts from a predefined photon beam source model and then performs downstream transport using a deterministic solution of the linear Boltzmann transport equation (9). In Monaco, the virtual source model contains primary photons, secondary photons, and electron contamination, together with parameters describing source size, fluence, energy spectrum, and angular source distribution; downstream dose transport is then calculated using the XVMC algorithm (10). Although the transport methods are different, both systems rely on an effective representation of the radiation source before the downstream dose calculation is performed. Thus, accurate downstream transport does not by itself remove sensitivity to the source representation used as input.

The Monaco source-modelling concept is also relevant to the present work. Its virtual-source characteristics are based on particle fluence, energy-fluence, and angular source distributions informed by BEAMnrc modelling of the linac treatment head (10). In the present study, BEAMnrc was also used to generate photon phase-space data from a linac-head simulation, and BEAMDP was then used to quantify the photon angular distribution before the phantom. The present work does not reproduce the Monaco virtual source model; it follows a related physical modelling principle in which detailed Monte Carlo head transport is used to characterize the effective photon field. Here, the angular distribution itself is treated as an observable and its sensitivity to primary electron source width is quantified.

In this paper, the terms *upstream* and *downstream* are used relative to the target and phantom. Upstream refers to accelerator processes that establish the electron-beam state before the target, including acceleration, bending, focusing, and steering. Downstream refers to photon production at the target and subsequent transport through the linac head toward the phantom. The phase-space plane analysed here is therefore downstream of target/head transport but upstream of the phantom. The current numerical study quantifies downstream consequences of a prescribed effective source change; it does not uniquely determine the upstream accelerator mechanism that generated that source state.

The upstream electron-beam state should not necessarily be regarded as perfectly fixed. Focal-spot and radiation-isocentre behaviour can vary with steering conditions, gantry angle, and time (11–13). Integrated accelerator simulations have also connected pre-target electron transport to the target electron distribution and subsequent photon dose, while transverse magnetic perturbations have been shown to modify the focal spot and downstream photon field (14, 15). These studies provide a physical basis for considering the Gaussian source used in Monte Carlo modelling as a reduced description of a more complex pre-target electron state. The present work does not claim that the broadened model M2 was produced by any specific steering, focusing, magnetic, filament-current, or RF mechanism.

The present work also considers a reduced statistical interpretation of an effective Monte Carlo source. In that interpretation, a stationary Gaussian source need not represent one immutable instantaneous electron distribution: it may instead represent the fluence-integrated projection of distinct instantaneous states whose centroids vary during irradiation. The prospective formulation separates intrinsic within-state source covariance from covariance of the source-state centroids. No time-resolved source dynamics are measured or reconstructed, and no accelerator-control variable is uniquely mapped to M1 or M2.

Photon phase space provides an intermediate physical level between the electron source and final dose distribution. Martins et al. showed that modification of particle energy and direction in a linac phase-space file can produce important changes in calculated depth-dose and lateral-dose profiles (16). Angular information in the photon phase space is therefore dosimetrically consequential. A change in the primary electron source can affect final dose not only through its spatial representation but also through changes in the photon angular field after target interaction and linac-head transport.

In the present study, source-width sensitivity means the change in a downstream observable produced by a prescribed change in the effective primary electron source width. It does not imply a measured source-width probability distribution or a clinical source-width uncertainty. The direct quantity is the endpoint difference between the two simulated source models. Measurement variability refers to the observed spread among detector/session data and is not interpreted as pure detector uncertainty or as a complete formal measurement uncertainty budget (4).

Recent time-aware radiotherapy frameworks make machine motion and delivery time explicit components of optimization rather than purely emergent properties of a fixed control-point representation. In particular, Li et al. introduced Dynamic Modulated Arc Therapy (DMAT), which jointly models delivery time, modulation, and machine-specific axis dynamics (17). That work concerns downstream treatment delivery and does not model the primary-electron source. Its relevance here is therefore prospective: it motivates the upstream question of whether an effective source phase space should ultimately remain strictly stationary or be conditioned on an evolving accelerator state. The present simulations do not answer that dynamic question; they establish the stationary endpoint-sensitivity baseline against which such a future model could be tested.

The term field-size amplification is also used in a specific sense. It does not mean that the primary electron source becomes broader when the radiation field becomes smaller. The same prescribed source-model change is applied at each field size. Field-size amplification means that the magnitude of the downstream response to this same source perturbation becomes larger as the field aperture is reduced.

Although previous studies have investigated detector effects, treatment-planning beam models, effective spot size, electron-source reconstruction, focal-spot behaviour, and phase-space optimization, the connection between primary electron source width, final pre-phantom photon angular phase space, and field-size-dependent sensitivity has not been examined in the same combined framework. It is also important to compare source-model sensitivity with the variability observed during practical measurement without treating both quantities as the same uncertainty source.

Therefore, the present study was restricted to the 6 MV photon beam and investigated two complementary parts of the small-field problem. First, empirical measurement variability was evaluated using five detector datasets for field sizes from 20×20 to 3×3 cm^2^. Second, Monte Carlo source-width sensitivity was evaluated for 3×3, 2×2, 1×1, and 0.5×0.5 cm^2^ fields using two primary electron source models. The Monte Carlo analysis included central-axis response, profile shape, penumbra, and photon angular phase-space quantities obtained from BEAMDP. The 3×3 cm^2^ field served as the overlap field where experimental measurement variability and source-model-induced profile shifts could be compared on the same relative-response scale. The main hypothesis was that the observable effect of the effective primary electron source becomes stronger as the radiation field becomes smaller.

## 2 Theory and formalism

### 2.1 Study framework and source-state definition

Two primary electron source models were considered. The narrow reference model was

(1) $$M1:\;{\text{FWHM}}_{x}={\text{FWHM}}_{y}=0.15\text{ cm},$$

whereas the broadened asymmetric model was

(2) $$M2:\;{\text{FWHM}}_{x}=0.35\text{ cm},\;{\text{FWHM}}_{y}=0.60\text{ cm}.$$

Throughout this study, the *x* direction is denoted inline and the *y* direction crossline. The two source configurations are endpoint scenarios rather than experimentally determined confidence limits of a clinical source-width distribution.

M1 was retained from our previous Monte Carlo study as the narrow reference source. In that study, the symmetric 0.15 cm FWHM was selected by averaging source-width values reported across several previous modelling studies (8). M2 was deliberately introduced in the same study as a broad asymmetric perturbation, with ${\text{FWHM}}_{x}=0.35\text{ cm}$ and ${\text{FWHM}}_{y}=0.60\text{ cm}$, specifically to test the dosimetric effect of a controlled departure from the idealized M1 source (8). The present analysis therefore treats M1 and M2 as prescribed sensitivity endpoints. M2 is not presented as a best-fit source, a confidence limit, or an independently measured clinical source distribution.

The demonstrated numerical chain is summarized in Equation 3 as follows:

(3) effective primary electron source→linac-head transport→photon angular phase space→dose/profile response.

### 2.2 Primary source-width sensitivity

For a generic observable *y*, the signed change produced by replacement of M1 by M2 is defined in Equation 4:

(4) $$\delta_{\text{source}}\left(y\right)=y\left(M2\right)-y\left(M1\right),$$

and the magnitude of the endpoint effect is

(5) $${\text{Δ}}_{\text{source}}\left(y\right)=\left|y\left(M2\right)-y\left(M1\right)\right|.$$

The directly simulated quantity is Δ_source_; no probability distribution is implied by (Equation 5).

### 2.3 Monte Carlo profile response

Let *D_M_*(*x*) denote the Monte Carlo profile ordinate for source model *M*. The central-axis, profile-shape, and penumbra quantities are defined in Equations 6–12. The common-scale central-axis ratio is

(6) $$R_{\text{CAX}}=\frac{D_{M2}\left(0\right)}{D_{M1}\left(0\right)},$$

and the percentage reduction under M2 is

(7) $${\text{Δ}}_{\text{CAX}}\left(\%\right)=100\left(1-R_{\text{CAX}}\right).$$

To examine profile shape independently of absolute central-axis amplitude,

(8) $$r_{M}\left(x\right)=\frac{D_{M}\left(x\right)}{D_{M}\left(0\right)},$$

with

(9) $$\delta_{\text{shape}}\left(x\right)=r_{M2}\left(x\right)-r_{M1}\left(x\right).$$

For a common physical scale preserving the amplitude change,

(10) $$r_{M}^{\left(M1\right)}\left(x\right)=\frac{D_{M}\left(x\right)}{D_{M1}\left(0\right)}.$$

### 2.4 Penumbra

The lateral field-edge response was characterized by the 20–80% penumbra,

(11) $$P_{20-80}=\left|x_{20\%}-x_{80\%}\right|,$$

with left and right values averaged as

(12) $${\bar{P}}_{20-80}=\frac{P_{20-80}^{L}+P_{20-80}^{R}}{2}.$$

### 2.5 Photon angular phase-space formalism

The photon angular phase-space quantities are defined in Equations 13–25. For a polar-angle bin *i*, let *W_i_* denote the summed photon statistical weight, *N*_inc_ the incident-primary count stored in the phase-space header, and ΔΩ*_i_* the annular solid angle of the bin,

(13) $${\text{ΔΩ}}_{i}=2\pi \left[\text{cos}\left(\theta_{i,\text{low}}\right)-\text{cos}\left(\theta_{i,\text{high}}\right)\right].$$

The BEAMDP-equivalent angular-density ordinate is

(14) $$y_{i}=\frac{W_{i}}{N_{\text{inc}}\;{\text{ΔΩ}}_{i}},$$

with units of particles per unit solid angle per incident primary. To reproduce the scalar angular-density convention used for the archived BEAMDP/Grace distributions, the normalized bin weight is

(15) $$p_{i}=\frac{y_{i}\text{Δ}\theta_{i}}{\sum_{j}y_{j}\text{Δ}\theta_{j}},\;\sum_{i}p_{i}=1.$$

For the nominal equally spaced histograms used here,

(16) $$\text{Δ}\theta =0.05^{\circ }.$$

Thus, *p_i_* is the normalized weight of the angular-density profile as a function of polar angle; it is not the raw annular particle probability $W_{i}/\sum_{j}W_{j}$. The same definition was used for all four field sizes. The mean, standard deviation, and true RMS angle are

(17) $$\bar{\theta }=\sum_{i}p_{i}\theta_{i},$$

(18) $$\sigma_{\theta }={\left[\sum_{i}p_{i}{\left(\theta_{i}-\bar{\theta }\right)}^{2}\right]}^{1/2},$$

(19) $$\theta_{\text{RMS}}={\left(\sum_{i}p_{i}\theta_{i}^{2}\right)}^{1/2},$$

with

(20) $$\theta_{\text{RMS}}^{2}=\sigma_{\theta }^{2}+{\bar{\theta }}^{2}.$$

The cumulative normalized angular-density distribution is

(21) $$F\left(\theta \right)=\sum_{\theta_{i}\leq \theta }p_{i},$$

and $q_{95}\equiv \theta_{95}$ satisfies

(22) $$F\left(\theta_{95}\right)=0.95.$$

The solid angle of the cone defined by this angular-density quantile is

(23) $$\Omega_{95}=2\pi \left(1-\text{cos}\theta_{95}\right),$$

with the small-angle approximation

(24) $${\text{Ω}}_{95}≃\pi \theta_{95}^{2}.$$

The high-angle angular-density fraction is

(25) $$T_{\theta \geq 1^{\circ }}=\sum_{\theta_{i}\geq 1^{\circ }}p_{i}.$$

Angular FWHM was not used as a primary scalar metric because the analysed polar-angle distributions are one-sided (*θ* ≥ 0).

### 2.6 Prospective effective-source interpretation

The Monte Carlo calculations reported in this study are stationary calculations using the prescribed M1 and M2 source-width endpoints; no time-dependent source transport is simulated. The dynamic interpretation is therefore reduced to the minimum statistical construction required to distinguish an instantaneous source state from a fluence-integrated effective source.

The effective-source and covariance relations are expressed in Equations 26–28. Let *p_n_*(r) denote a normalized instantaneous transverse source distribution and *w_n_* its fluence fraction, with $\sum_{n}w_{n}=1$. The fluence-integrated effective source is

(26) $$p_{\text{eff}}\left(r\right)=\sum_{n}w_{n}p_{n}\left(r\right).$$

If Σ_inst_ denotes the within-state covariance and *C_μ_* the covariance of the state centroids, the law of total covariance gives

(27) $$\Sigma_{\text{eff}}={\langle \Sigma_{\text{inst}}\rangle }_{w}+C_{\mu }.$$

For a one-dimensional special case with a common intrinsic width σ_0_ and source-centroid coordinate *X_s_*,

(28) $$\sigma_{\text{eff}}^{2}=\sigma_{0}^{2}+{\text{Var}}_{w}\left(X_{s}\right).$$

Thus, a broader stationary effective source may in principle reflect intrinsic source broadening, unresolved centroid-state variability, or both. The present study does not determine this decomposition and does not identify either mechanism as the origin of M2. M1 and M2 remain prescribed stationary sensitivity endpoints.

### 2.7 Literature-derived transverse steering-to-focal-spot response coefficient

The steering-response coefficient and its literature-based reconstruction are presented in Equations 29 and 30. For prospective hypothesis construction, a local transverse steering-to-focal-spot position response coefficient is defined as

(29) $$c_{T}\equiv \frac{\partial x_{f}}{\partial I_{2T}},\;\left[c_{T}\right]=\text{mm}/\text{mA},$$

where *x_f_* is transverse focal-spot position and *I*_2_*_T_* is the transverse 2T steering current. Programmatic digitization of the five published transverse 2T settings of Chojnowski et al. (12) gives a direct local least-squares slope $c_{T}^{\left(\text{direct}\right)}\approx -0.01977$ mm/mA. An independent reconstruction within the same published dataset uses the current-to-symmetry slope $\partial S/\partial I_{2T}\approx -0.1741$ %/mA together with the reported focal-spot-to-symmetry response $\partial S/\partial x_{f}=8.6$ %/mm, giving

(30) $$c_{T}^{\left(\text{symmetry}\right)}=\frac{\partial S/\partial I_{2T}}{\partial S/\partial x_{f}}\approx -0.02024\text{ mm}/\text{mA}.$$

The magnitudes differ by approximately 2.35%, motivating the rounded local scale $\left|c_{T}\right|\approx 0.020$ mm/mA. This quantity is a figure-derived local response coefficient from the published Chojnowski accelerator, not a manufacturer calibration, a universal Elekta constant, or a calibration of the accelerator used for the present measurements. The sign is coordinate- and current-convention dependent.

As an external order-of-scale check at the current-to-beam-response level, van Appeldoorn et al. reported an example 2T steering-current-to-tilt gradient of −0.134%/mA from Elekta service-graph data (18). This is not a second independent measurement of *c_T_* in mm/mA and is not converted into one here. Their later work further demonstrated the practical use of measured 2R/2T service-graph steering information for gantry-dependent beam-control optimization (19).

A future state-conditioned model could test the local linear relation $\delta x_{f}\left(t\right)≃c_{T}\;\delta I_{2T}\left(t\right)$ and, only if a time-resolved steering-current distribution were independently established, $\text{Var}\left(x_{f}\right)≃c_{T}^{2}\text{Var}\left(I_{2T}\right)$. The proposed steering-response and variance framework is conceptually related to established beam-orbit measurement and correction methods (20). No temporal $I_{2T}\left(t\right)$ series was acquired in the present study, no steering-current variance is inferred, and no inverse current requirement is reported as a result.

## 3 Measurements and methodology

The experimental field definitions, normalized-response and variability measures, and simulated field settings are specified in Equations 31–37.

### 3.1 Experimental setup

The experimental part of this study was performed using the 6 MV flattened photon beam of a clinical Elekta Versa HD linear accelerator. Measurements were acquired in an RW3 slab phantom at a source-to-surface distance (SSD) of 100 cm and at a depth of 10 cm. A 10×10 cm^2^ field was used as the reference field.

Detector readings were obtained using an IBA DOSE 2 electrometer. The appropriate absorbed-dose-to-water calibration coefficient, $N_{D,w,Q_{0}}$, and beam-quality correction factor, $k_{Q,Q_{0}}$, were applied for each detector according to its calibration data. Individual uncertainty contributions of the calibration and correction factors were not separately propagated because the experimental arm was designed to retain the combined variability observed in the practical measurement process.

Measurements were obtained in both inline and crossline directions at selected off-axis positions extending from the central region toward the geometric field boundary. The field sizes retained for the experimental analysis were

(31) $$3\times 3,\;5\times 5,\;10\times 10,\;20\times 20\;{\text{cm}}^{2}.$$

Only 6 MV data were included in the present analysis.

### 3.2 Detectors

Five detector datasets were analysed: CC04, CC13, IBA FC65-G Farmer-type ionization chamber, PTW 30001 Farmer-type ionization chamber, and IBA PPC05 parallel-plate ionization chamber. The detectors have different cavity geometries, sensitive volumes, and response characteristics. Their purpose here was not to identify a single detector as the true reference at all field sizes, but to characterize variability that can appear during practical measurements.

The datasets were acquired during separate measurement periods. The observed spread may therefore include detector response, setup and repositioning, environmental conditions, temporal beam variation, and operator-related effects. It is consequently described as empirical measurement variability or inter-detector/session measurement variability, not as pure detector uncertainty or Type-A repeatability uncertainty.

### 3.3 Experimental normalization and variability analysis

Because the five detectors have different absolute sensitivities, each detector was normalized to its own central-axis reading. For detector *c*,

(32) $$r_{c}\left(x\right)=\frac{M_{c}\left(x\right)}{M_{c}\left(0\right)},$$

where *M_c_*(*x*) is the detector reading at off-axis position *x* and *M_c_*(0) is the central-axis reading from the same detector.

For *n* = 5 detectors, the mean relative response was

(33) $$\bar{r}\left(x\right)=\frac{1}{n}\sum_{c=1}^{n}r_{c}\left(x\right),$$

and the empirical spread was quantified using the sample standard deviation

(34) $$s_{\text{meas}}\left(x\right)=\sqrt{\frac{\sum_{c=1}^{n}{\left[r_{c}\left(x\right)-\bar{r}\left(x\right)\right]}^{2}}{n-1}}.$$

The coefficient of variation was

(35) $$CV\left(x\right)=100\frac{s_{\text{meas}}\left(x\right)}{\bar{r}\left(x\right)}.$$

No attempt was made to decompose the observed spread into independent detector, setup, temporal, environmental, or operator components.

### 3.4 Normalized-position control of the experimental field-size comparison

To test whether the field-size trend in empirical inter-detector/session variability was influenced by non-equivalent spatial sampling, each off-axis measurement position was also expressed as the normalized coordinate

(36) $$\xi =\frac{\left|x\right|}{F/2},$$

where *F* is the nominal square-field side length. The available datasets contained *ξ* = 1.0 at the geometric field boundary for all evaluated field sizes, whereas interior normalized positions were not common to every field. Cross-field matched-position analysis was therefore restricted to *ξ* = 1. Aggregate field means were retained as descriptive summaries of all sampled positions but were not interpreted as spatially controlled field-size effects.

### 3.5 Monte Carlo model of the linear accelerator

The Monte Carlo calculations used a generalized Elekta Versa HD model with an Agility treatment head, derived from the previously published model and public literature (8, 21, 22). The Agility MLC contains 160 leaves arranged in two opposing banks, with a nominal projected leaf width of 0.5 cm at isocenter. The current study was restricted to 6 MV-FF.

The two source models were those defined in (Equations 1 and 2). The Monte Carlo fields were

(37) $$0.5\times 0.5,\;1\times 1,\;2\times 2,\;3\times 3\;{\text{cm}}^{2}.$$

The 3×3 cm^2^ field served as the experimental-Monte Carlo overlap field.

### 3.6 EGSnrc and BEAMnrc treatment-head simulation

Simulations were performed with the EGSnrc Monte Carlo system (23). BEAMnrc was used to simulate electron and photon transport through the linac treatment head and to generate particle phase-space files (24, 25). The incident primary-electron beam was modelled as monoenergetic at 6.7 MeV, originating from the *z* = 0 source plane with zero initial angular divergence. The previously published production protocol was configured for 2×10^8^ primary histories per field/source-model configuration (8). Each field/source-model combination was simulated in a separate BEAMnrc run and generated its own phase-space output; no phase-space file was shared between different configurations. All source and transport settings were kept identical across field sizes, with only the jaw/MLC aperture definition changed to generate the corresponding nominal field size. The complete jaw and MLC aperture definitions are provided in the archived BEAMnrc input files. Particles were scored at 100 cm from the source at the final pre-phantom scoring plane. Electron and photon transport cutoffs were *ECUT* = 0.7 MeV and *PCUT* = 0.01 MeV, respectively. Electron range rejection was applied with a 2 MeV threshold, and uniform bremsstrahlung splitting was used as the BEAMnrc variance-reduction technique. The 700 ICRU dataset was used for transport calculations. For the angular-density analysis, *N*_inc_ was read from the incident-primary count stored in each archived phase-space header, matching the normalization used in the BEAMDP-equivalent reconstruction. The previously generated calculations were performed using the Bibliotheca Alexandrina Supercomputer Facility, Alexandria, Egypt (8).

### 3.7 DOSXYZnrc dose calculation

The BEAMnrc phase-space files were used as incident photon sources for dose calculations with DOSXYZnrc (26). DOSXYZnrc transports particles through a three-dimensional rectilinear voxel phantom and calculates deposited dose. For the present comparisons, the DOSXYZnrc values are reported as dose per incident primary history under the same Monte Carlo normalization for M1 and M2. Each corresponding DOSXYZnrc dose calculation was performed separately using 1.1×10^10^ histories. The full BEAMnrc phase-space file was used as the photon source without phase-space particle recycling (recycling number = 0) or additional photon splitting (photon splitting number = 0). Global transport cutoffs were *ECUT* = 0.7 MeV and *PCUT* = 0.01 MeV, and range rejection was enabled with *ESAVE* = 2 MeV. Dose was scored in a 30×30×30 cm^3^ water phantom extending from −15 to +15 cm in both lateral directions and from 0 to 30 cm in depth, using voxel dimensions of 0.24×0.24×0.50 cm^3^. The 20–80% penumbra and *W*_50_ crossing positions were determined by linear interpolation between adjacent sampled profile points bracketing the corresponding normalized-dose levels. The history count yielded statistical dose uncertainties below approximately 0.5% in the original calculations (8). The present analysis used the calculated profiles to evaluate central-axis response, field-edge response, self-normalized shape, common-reference response, and 20–80% penumbra. Monte Carlo statistical uncertainty was retained as a separate quantity and was not included in the definition of Δ_source_.

### 3.8 BEAMDP angular phase-space analysis

The BEAMnrc phase-space files were separately processed using BEAMDP (27). The analysis extracted photon angular distributions at the same final pre-phantom scoring plane. These distributions represent the photon angular phase space after target interaction and treatment-head transport; they are not isolated target-emission distributions.

The angular distributions were analysed for 0.5×0.5, 1×1, 2×2, and 3×3 cm^2^ fields for both M1 and M2. The nominal bin width was 0.05°. The derived observables were $\bar{\theta }$, σ*_θ_*, *θ*_RMS_, *q*_95_, Ω_95_, and $T_{\theta \geq 1^{\circ }}$, using the definitions above.

### 3.9 Directional projected-angle phase-space analysis

The projected-angle definitions and computational workflow are summarized in Equations 38–41. Because M2 was anisotropic, the scalar polar-angle analysis was complemented by projected photon-angle distributions. For each photon with transverse direction cosines *u* and *v* and downstream longitudinal cosine *w*, the projected angles were defined as:

(38) $$\theta_{x}=\text{atan}2\left(u,w\right),\;\theta_{y}=\text{atan}2\left(v,w\right).$$

The *x* and *y* directions correspond to the inline and crossline axes, respectively. Directional containment was characterized from $\left|\theta_{x}\right|$ and $\left|\theta_{y}\right|$ using *q*_80_, *q*_90_, and *q*_95_, while the central signed-angle distributions were additionally summarized by their FWHM. Directional percentile precision was evaluated with the same history-preserving grouped resampling strategy used for the revised scalar angular-statistics analysis. These projected metrics resolve directional redistribution but do not isolate independent *x*-only and *y*-only source perturbations because both M2 source widths were changed simultaneously.

### 3.10 Complementary roles of BEAMnrc, DOSXYZnrc, and BEAMDP

The three EGSnrc tools had complementary roles. BEAMnrc simulated the linac-head transport and generated the particle phase space. DOSXYZnrc transported this phase-space source into the phantom and calculated dose. BEAMDP analysed particle information already stored in the phase-space files, with emphasis on photon angular divergence. The workflow can be summarized as

(39) $$\text{primary electron source}\overset{\text{BEAMnrc}}{⟶}\text{pre-phantom photon phase space},$$

followed by either

(40) $$\text{phase space}\overset{\text{BEAMDP}}{⟶}\text{angular observables},$$

or

(41) $$\text{phase space}\overset{\text{DOSXYZnrc}}{⟶}\text{dose/profile observables}.$$

This structure is conceptually related to virtual-source approaches in which detailed head transport is used to characterize an effective radiation source, but the present study does not reproduce the clinical Monaco VSM (10).

### 3.11 Python-based computational post-processing

Custom Python scripts were used for numerical post-processing of the Monte Carlo phase-space analysis. The BEAMDP-derived angular histograms were imported and processed programmatically to normalize angular-bin weights and calculate the mean angle, standard deviation, true RMS angle, *q*_95_, Ω_95_, and the high-angle tail fraction according to the angular definitions above. The scripts were also used for numerical M1/M2 comparisons and generation of the derived tables and plots reported from the phase-space study.

For the revised phase-space analysis, additional custom Python code was developed to read the archived BEAMnrc phase-space files directly, identify primary-history boundaries from the stored history markers, and extract the particle-level quantities required for the scalar and directional angular analyses. The code reconstructed polar and projected photon angles from the recorded direction cosines, preserved all particles from a common primary history within the same statistical unit, generated the grouped Poisson bootstrap resamples, repeated the angular-bin-width sensitivity analysis, and produced the derived statistical summaries used in the revised tables and figures.

Statistical precision of the angular observables was re-evaluated directly from the archived BEAMnrc phase-space records using the primary-history markers retained in the phase-space format. Particles generated by the same incident primary history were kept within the same statistical unit so that within-history correlations were preserved. For uniform processing of the eight phase-space files, intact primary histories were assigned to 1,000 interleaved, non-overlapping history groups, and 20,000 Poisson bootstrap replicates were generated by applying independent Poisson(1) multiplicities to those groups. The angular histogram and the derived *q*_95_, Ω_95_, and $T_{\theta \geq 1^{\circ }}$ values were recomputed for each replicate. The standard deviation of the bootstrap distribution was reported as the 1σ statistical uncertainty and the 2.5th–97.5th percentiles as the bootstrap 95% interval. The grouped implementation was cross-checked against direct individual-history resampling for the 0.5×0.5 and 1×1 cm^2^ files, with uncertainty estimates agreeing within 5.4% for $q_{95}/{\text{Ω}}_{95}$ and within 4.5% for the high-angle-tail uncertainty. This history-preserving resampling estimates finite-history precision conditional on the archived phase-space realization and is not equivalent to newly generated independent BEAMnrc transport replicas. Angular-bin-width sensitivity was additionally evaluated by repeating the analysis at 0.025°, 0.05°, and 0.10°. Ajaz et al. (28) is cited as an example of independent-replica Monte Carlo uncertainty assessment, while the history-by-history statistical treatment in the BEAM code system is supported by Walters et al. (29).

Python was used for phase-space parsing, data extraction, numerical derivation, uncertainty analysis, and visualization; it was not used as a substitute for the EGSnrc/BEAMnrc particle-transport simulations.

### 3.12 Experimental-Monte Carlo overlap field

The experimental and Monte Carlo datasets intentionally covered different field-size domains. Experimental variability was evaluated from 20×20 down to 3×3 cm^2^, whereas source-width sensitivity was evaluated at 3×3, 2×2, 1×1, and 0.5×0.5 cm^2^. The 3×3 cm^2^ field was the methodological bridge. At this field, both experimental and Monte Carlo profiles could be expressed as center-normalized relative responses, allowing a direct descriptive comparison of *s*_meas_ and Δ_source_. The 2×2, 1×1, and 0.5×0.5 cm^2^ cases were Monte Carlo endpoint-sensitivity analyses only; no corresponding sub-centimeter experimental validation was performed in the present study.

### 3.13 Scope of the Monte Carlo model and clinical applicability

The experimental measurements were acquired on a clinical Elekta Versa HD. The Monte Carlo model, however, was not constructed as a machine-specific digital replica of that individual accelerator. It was a generalized Versa HD/Agility representation based on published information and previously published modelling work (8). No confidential or proprietary manufacturer information and no site-specific internal machine geometry or commissioning data were used to define the Monte Carlo treatment-head or primary-source models.

The use of the Elekta Versa HD and Agility names therefore identifies the general accelerator and treatment-head class represented by the simulation; it does not imply that the model geometry or source parameters were supplied, approved, or validated by the manufacturer. Likewise, M1 and M2 are controlled numerical source states and should not be interpreted as measurements of the actual source state of the clinical accelerator used for measurement.

The experimental and Monte Carlo datasets are used as complementary datasets rather than as a machine-matching or digital-twin validation exercise. The measurements quantify empirical measurement variability, whereas the simulations isolate source-model sensitivity under controlled numerical conditions. The Monte Carlo results are intended for theoretical and methodological sensitivity analysis and should not be directly applied to clinical treatment planning, commissioning, or machine-specific QA without independent local validation.

### 3.14 Reference M1 baseline benchmarking

The narrow M1 source was additionally benchmarked against an independently acquired 6 MV 10×10 cm^2^ commissioning percentage-depth-dose (PDD) curve measured with a CC13 chamber. The measured and Monte Carlo curves were independently normalized to their respective *d*_max_ values. Quantitative comparison was performed at the Monte Carlo depth points for *z* ≥ 2 cm after linear interpolation of the commissioning PDD. A descriptive ±2 percentage-point comparison band was used to summarize the agreement; this benchmark is a plausibility check of the reference M1 beam and is not presented as a machine-specific digital-twin validation. The previously published study also reported small-field output-factor comparisons between the M1/M2 calculations and Razor-diode/CC04 measurements (8).

## 4 Results

### 4.1 Experimental 6 MV measurement variability

Experimental variability showed a pronounced field-size dependence. The mean inter-detector coefficient of variation increased progressively as the field decreased from 20×20 to 3×3 cm^2^ (**Table 1**; **Figure 1**).

**Table 1:** Field-size dependence of empirical 6 MV measurement variability across the five chamber datasets.

Field size (cm^2^)	Mean CV (%)	Median CV (%)	Maximum CV (%)
20×20	2.46	1.55	8.62
10×10	4.05	1.86	16.29
5×5	9.50	5.15	26.28
3×3	15.45	15.34	26.42

**Figure 1:**
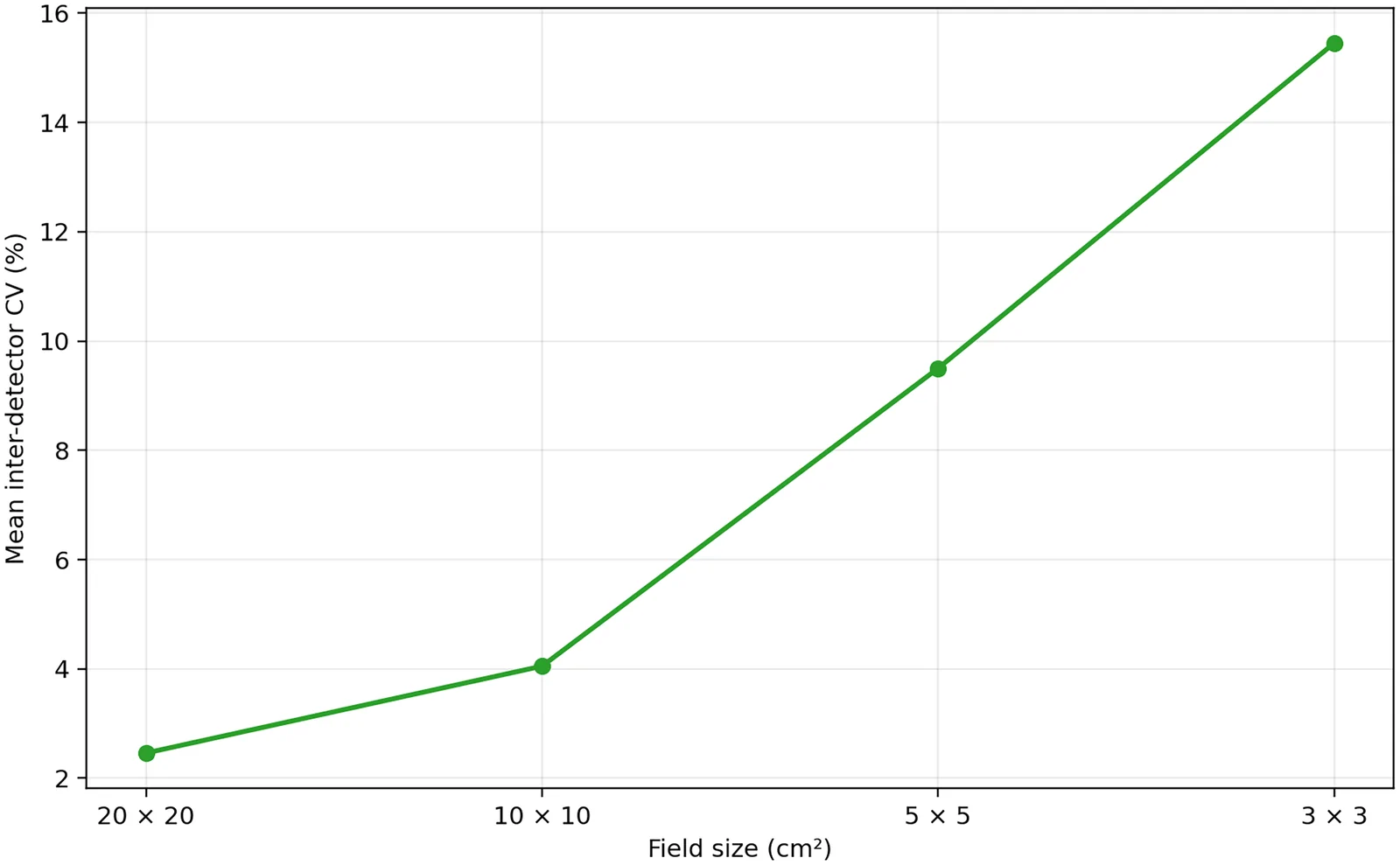
Mean 6 MV inter-detector coefficient of variation versus field size. Only the five chamber datasets included in the present analysis are represented.

The 3×3 cm^2^ field showed marked axis dependence (**Table 2**). Inline edge CV values were 26.42% and 24.65%, whereas crossline values were 4.71% and 6.03%.

**Table 2:** Empirical 6 MV measurement variability at the 3×3 cm^2^ field boundary.

Position	Mean relative response	*s*_meas_	CV (%)	Range across detectors
Inline +1.5	0.81361	0.21496	26.42	0.57933–1.07101
Inline −1.5	0.61486	0.15159	24.65	0.35668–0.72464
Crossline +1.5	0.66505	0.03130	4.71	0.62736–0.69936
Crossline −1.5	0.74913	0.04519	6.03	0.70607–0.81395

Position dependence was also observed in the larger fields, with edge positions generally showing larger variability than points well inside the field (**Table 3**).

**Table 3:** Representative position dependence of empirical 6 MV measurement variability in the larger fields.

Field	Sampling region	Observed mean CV
5×5	± 1.5 cm points	3.20%
5×5	± 2.5 cm edge points	15.79%
10×10	± 1.5 cm	1.75%
10×10	± 2.5 cm	1.12%
10×10	± 5 cm edge	9.27%
20×20	± 1.5 cm	1.63%
20×20	± 10 cm edge	3.49%

### 4.2 Matched normalized-position experimental comparison

The aggregate sampled-position mean CV values remained 2.46%, 4.05%, 9.50%, and 15.45% for 20×20, 10×10, 5×5, and 3×3 cm^2^, respectively. The sampled normalized positions, however, differed by field size: *ξ* = 1.0 for 3×3; 0.60 and 1.0 for 5×5; 0.30, 0.50, and 1.0 for 10×10; and 0.15, 0.25, 0.50, and 1.0 for 20×20 cm^2^. Because *ξ* = 1 was the only normalized position common to all four fields, the matched cross-field comparison was performed at the field boundary.

At *ξ* = 1, the inline boundary CV increased monotonically from 5.33% to 25.54% as field size decreased, whereas the crossline values were 1.66%, 2.95%, 8.19%, and 5.37%. The combined boundary mean increased strongly toward small fields but was not strictly monotonic between 5×5 and 3×3 cm^2^. The experimental variability is therefore described as field-size-, position-, and axis-dependent.

### 4.3 Reference M1 baseline benchmark

For the 10×10 cm^2^ reference field, the M1 Monte Carlo PDD showed close quantitative agreement with the CC13 commissioning PDD after independent normalization to *d*_max_. For *z* ≥ 2 cm, the mean absolute difference was 0.97 percentage point, the RMS difference was 1.04 percentage points, and the maximum absolute difference was 1.68 percentage points. All 56 evaluated Monte Carlo depth points were within ±2 percentage points of the interpolated commissioning curve. The measured and calculated *d*_max_ values were 1.50 and 1.75 cm, respectively; the 2.5 mm difference is comparable to one-half of the 5 mm Monte Carlo depth sampling interval. The normalized comparison is shown in **Supplementary Figure S1**. The previously published study provides additional supporting output-factor comparisons with Razor-diode and CC04 measurements (8).

### 4.4 Monte Carlo profile sensitivity between the M1 and M2 source endpoints

The common-scale central-axis response showed strong field-size-dependent endpoint sensitivity, but the response was not strictly monotonic across all four fields (**Table 4**; **Figure 2**). The tabulated CAX values are DOSXYZnrc dose per incident primary history under the same Monte Carlo normalization for M1 and M2.

**Table 4:** Central-axis DOSXYZnrc dose per incident primary history for the two source models under a common Monte Carlo normalization.

Field	D_M1_ (0)	D_M2_ (0)	R_CAX_	M2 reduction	Relative MC error (M1/M2)
0.5 × 0.5 cm^2^	1.5143 × 10^−16^	6.6110×10^−17^	0.43657	56.34%	0.308%/0.358%
1 × 1 cm^2^	1.7140 × 10^−16^	1.2068×10^−16^	0.70408	29.59%	0.310%/0.265%
2 × 2 cm^2^	1.7453 × 10^−16^	1.6010×10^−16^	0.91732	8.27%	0.264%/0.232%
3 × 3 cm^2^	1.7800 × 10^−16^	1.6291×10^−16^	0.91523	8.48%	0.234%/0.328%

**Figure 2:**
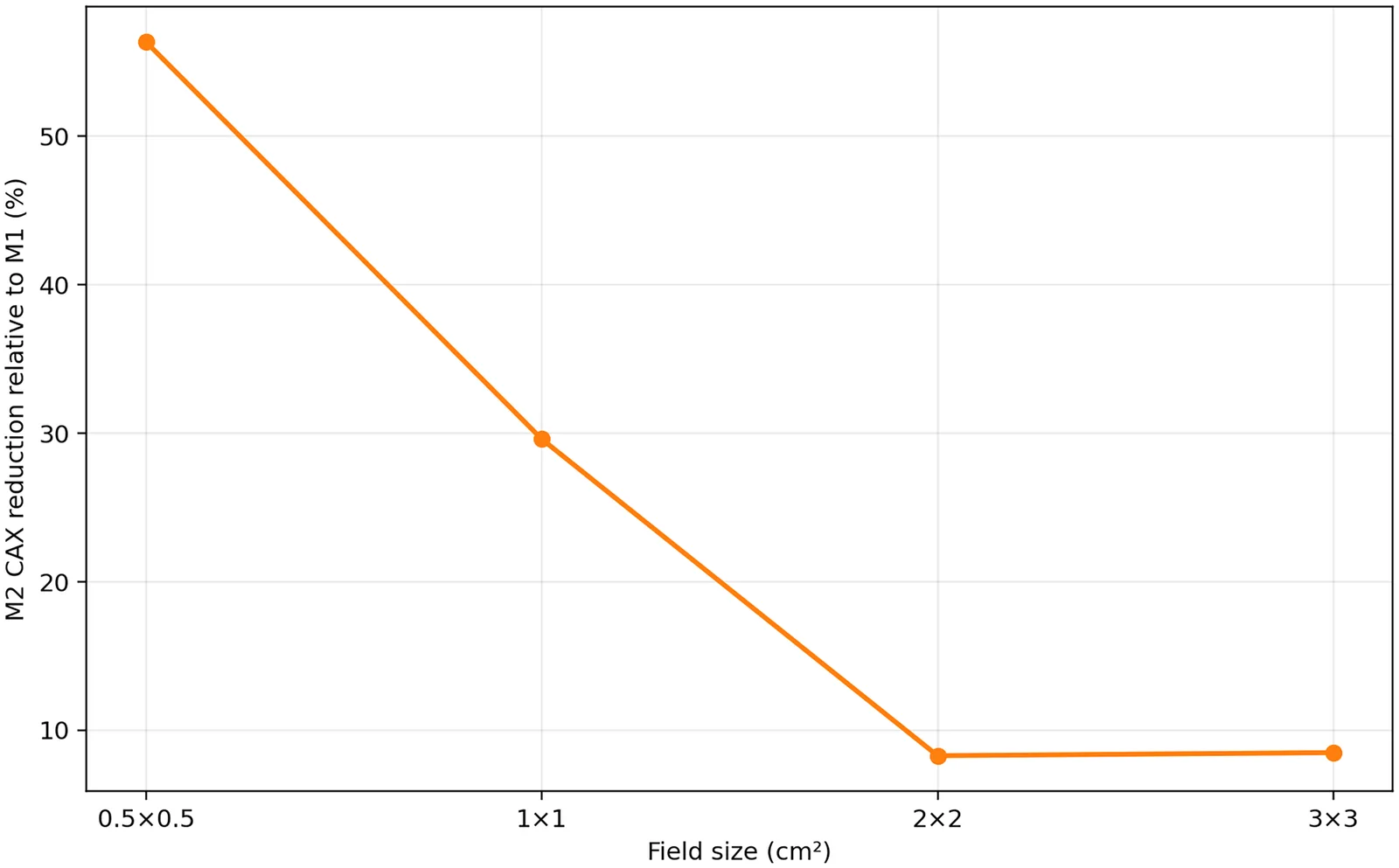
Reduction of the M2 central-axis response relative to M1 across the four Monte Carlo fields. The CAX response is not strictly monotonic; the strongest amplification occurs below 2×2 cm^2^.

The M2 response was reduced relative to M1 by 8.48% at 3×3, 8.27% at 2×2, 29.59% at 1×1, and 56.34% at 0.5×0.5 cm^2^. The near-equality of the 3×3 and 2×2 reductions shows that CAX amplification becomes pronounced below 2×2 cm^2^ rather than following a universal monotonic four-field law. The reported Monte Carlo statistical uncertainties were approximately 0.20–0.38%, substantially smaller than these endpoint differences.

The 20–80% penumbra increased under M2 for every field and axis (**Table 5**; **Figure 3**). The 50% widths are included in **Table 5** for completeness but do not show one monotonic field-size trend.

**Table 5:** Mean 20–80% penumbra and 50% field width for both source models.

Field	Axis	P_20–80_ M1 (cm)	M2 (cm)	Increase	W_50_ M1 (cm)	M2 (cm)
0.5×0.5	Inline	0.3258	0.4621	41.9%	0.6818	0.9451
0.5×0.5	Crossline	0.2905	0.4425	52.3%	0.6582	0.9481
1×1	Inline	0.3735	0.5648	51.2%	1.1917	1.2644
1×1	Crossline	0.2679	0.5272	96.8%	1.1744	1.3161
2×2	Inline	0.3717	0.6173	66.1%	2.2730	2.2994
2×2	Crossline	0.3150	0.6071	92.7%	2.2568	2.2806
3×3	Inline	0.4343	0.6421	47.9%	3.4056	3.3972
3×3	Crossline	0.3908	0.6145	57.3%	3.4120	3.4005

**Figure 3:**
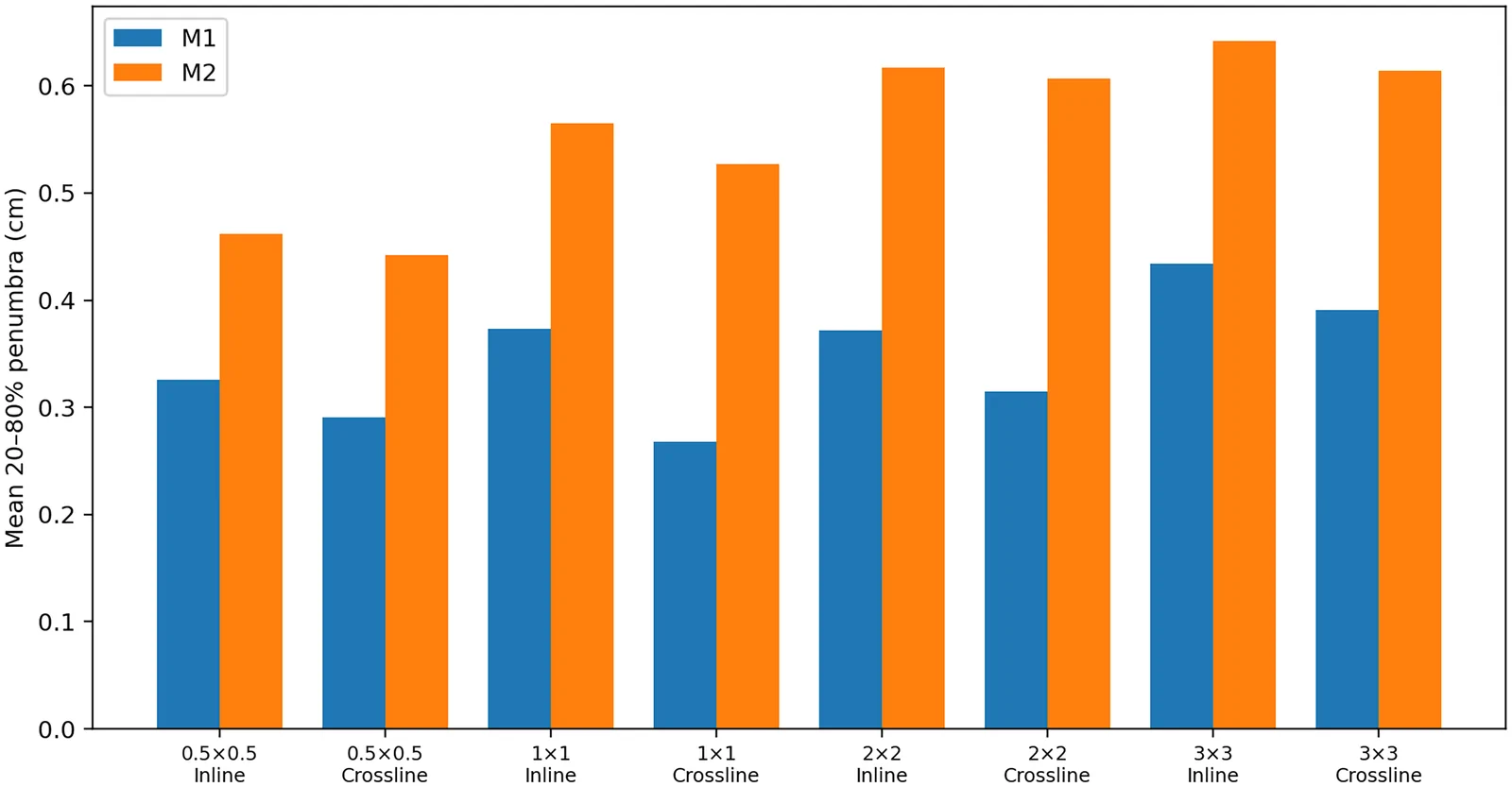
Mean 20–80% penumbra for M1 and M2. The broadened source increases penumbra in every evaluated field and axis.

Self-normalized profile-shape differences were not monotonic (**Table 6**; **Figures 4**–**7**). In the 0.5×0.5 cm^2^ field, the M2 edge-to-center ratio became larger because the M2 central-axis value decreased more strongly than the edge response.

**Table 6:** Self-normalized field-edge response. Differences are M2 − M1.

Field	Axis	M1 − edge	M2 − edge	Difference	M1 + edge	M2 + edge	Difference
0.5×0.5	Inline	0.6854	0.8178	+0.1324	0.6957	0.8206	+0.1249
0.5×0.5	Crossline	0.6852	0.8258	+0.1406	0.6880	0.8264	+0.1384
1×1	Inline	0.6879	0.6605	-0.0274	0.6937	0.6635	-0.0302
1×1	Crossline	0.7157	0.7034	-0.0122	0.7143	0.7062	-0.0081
2×2	Inline	0.7577	0.6654	-0.0922	0.7601	0.6629	-0.0972
2×2	Crossline	0.7995	0.6537	-0.1458	0.7981	0.6530	-0.1451
3×3	Inline	0.7538	0.7032	-0.0506	0.7624	0.7049	-0.0575
3×3	Crossline	0.7802	0.7134	-0.0668	0.7834	0.7094	-0.0740

**Figure 4:**
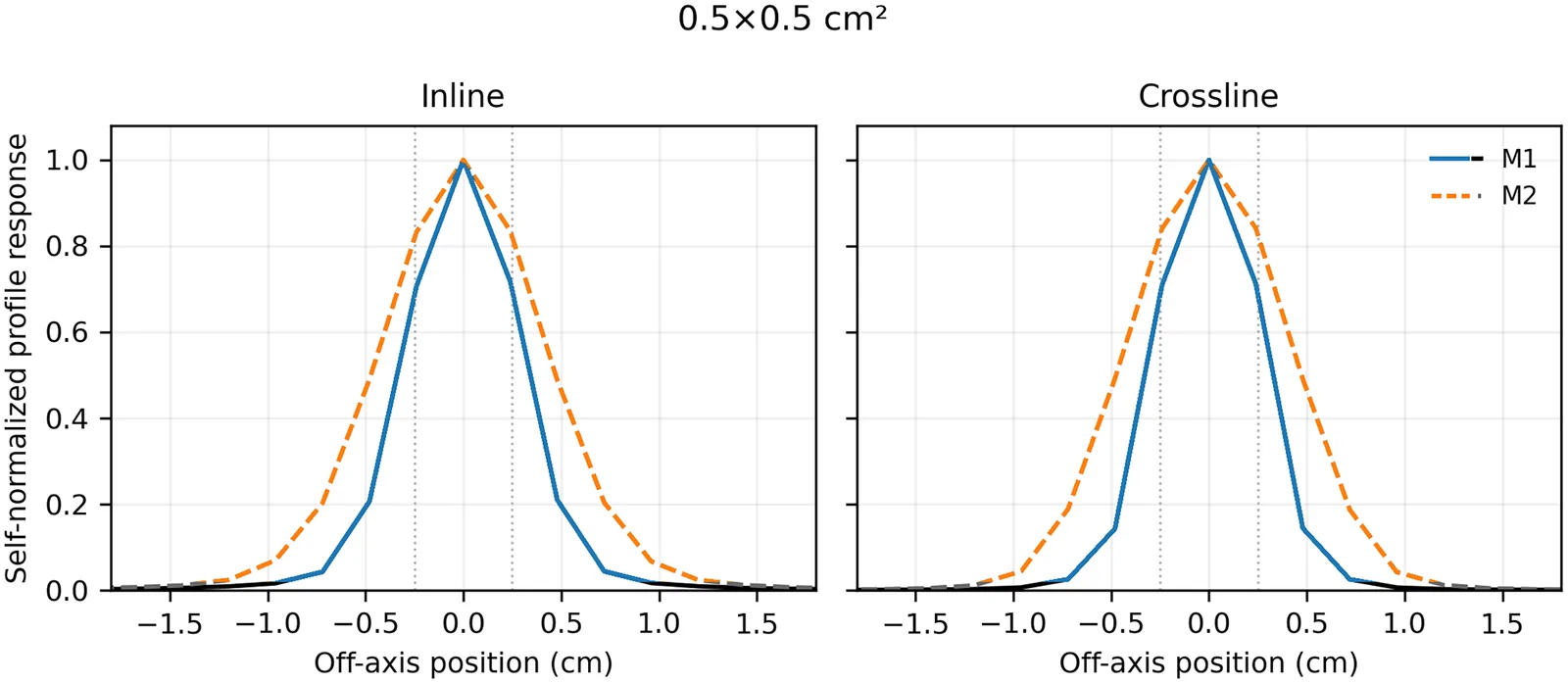
Self-normalized 0.5 × 0.5 cm^2^ Monte Carlo profiles for M1 and M2: left panel, inline; right panel, crossline. The light-gray dotted vertical lines indicate the nominal field boundaries.

**Figure 5:**
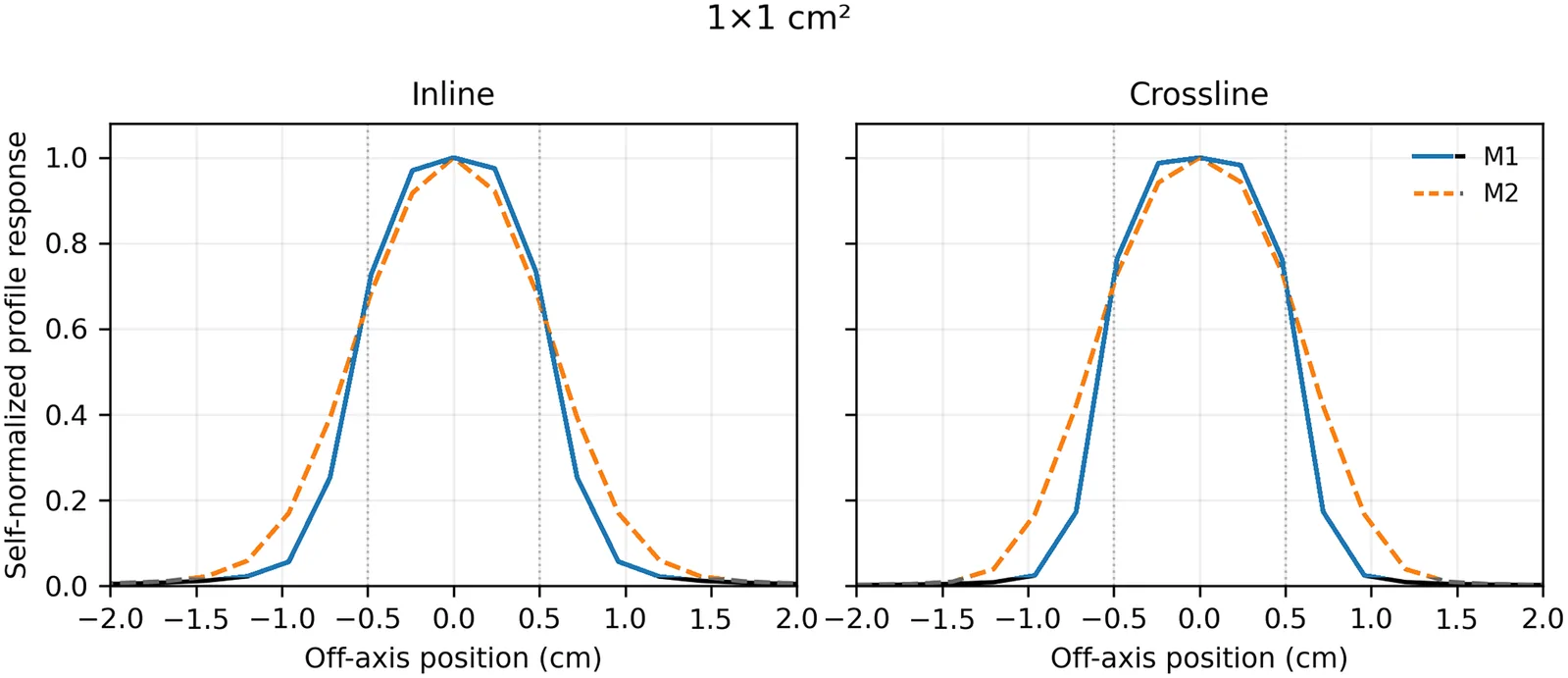
Self-normalized 1 × 1 cm^2^ Monte Carlo profiles for M1 and M2: left panel, inline; right panel, crossline. The light-gray dotted vertical lines indicate the nominal field boundaries.

**Figure 6:**
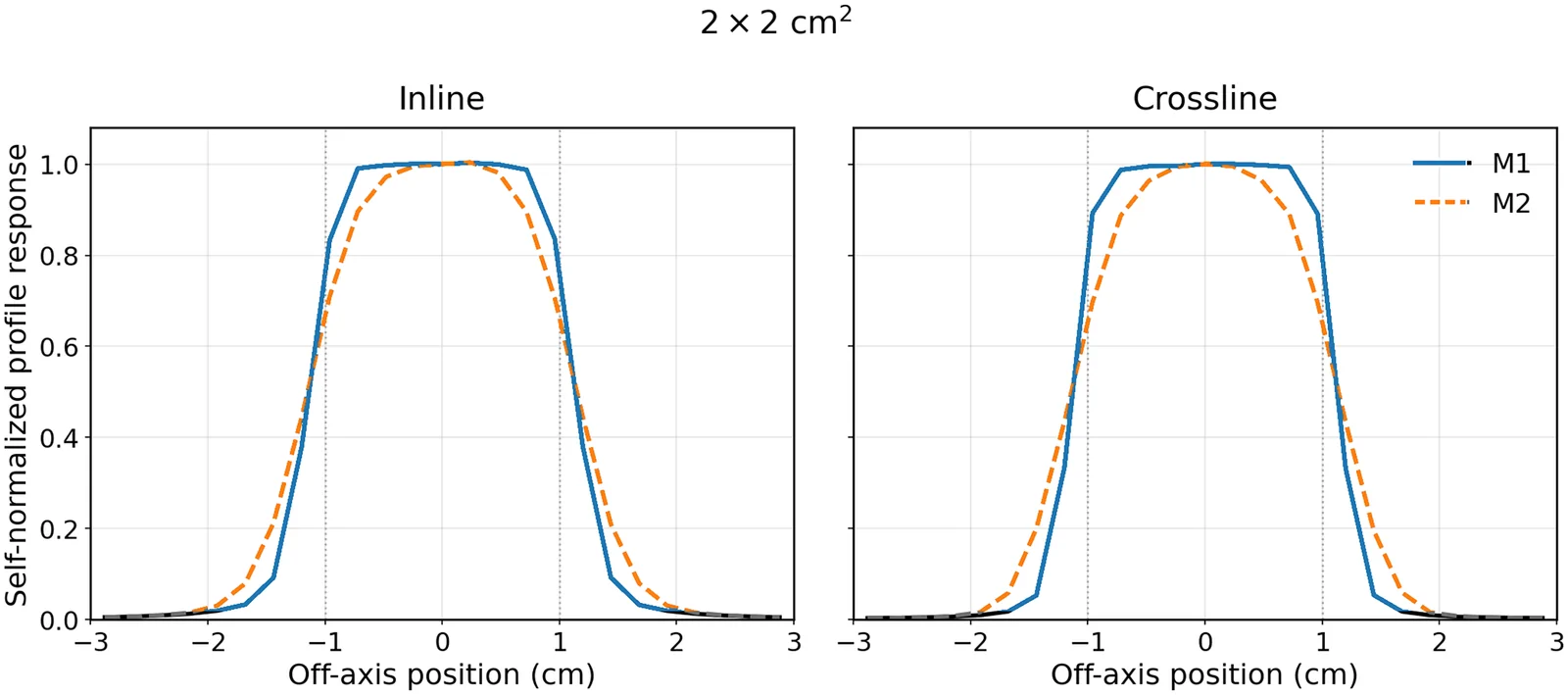
Self-normalized 2 × 2 cm^2^ Monte Carlo profiles for M1 and M2: left panel, inline; right panel, crossline. The light-gray dotted vertical lines indicate the nominal field boundaries.

**Figure 7:**
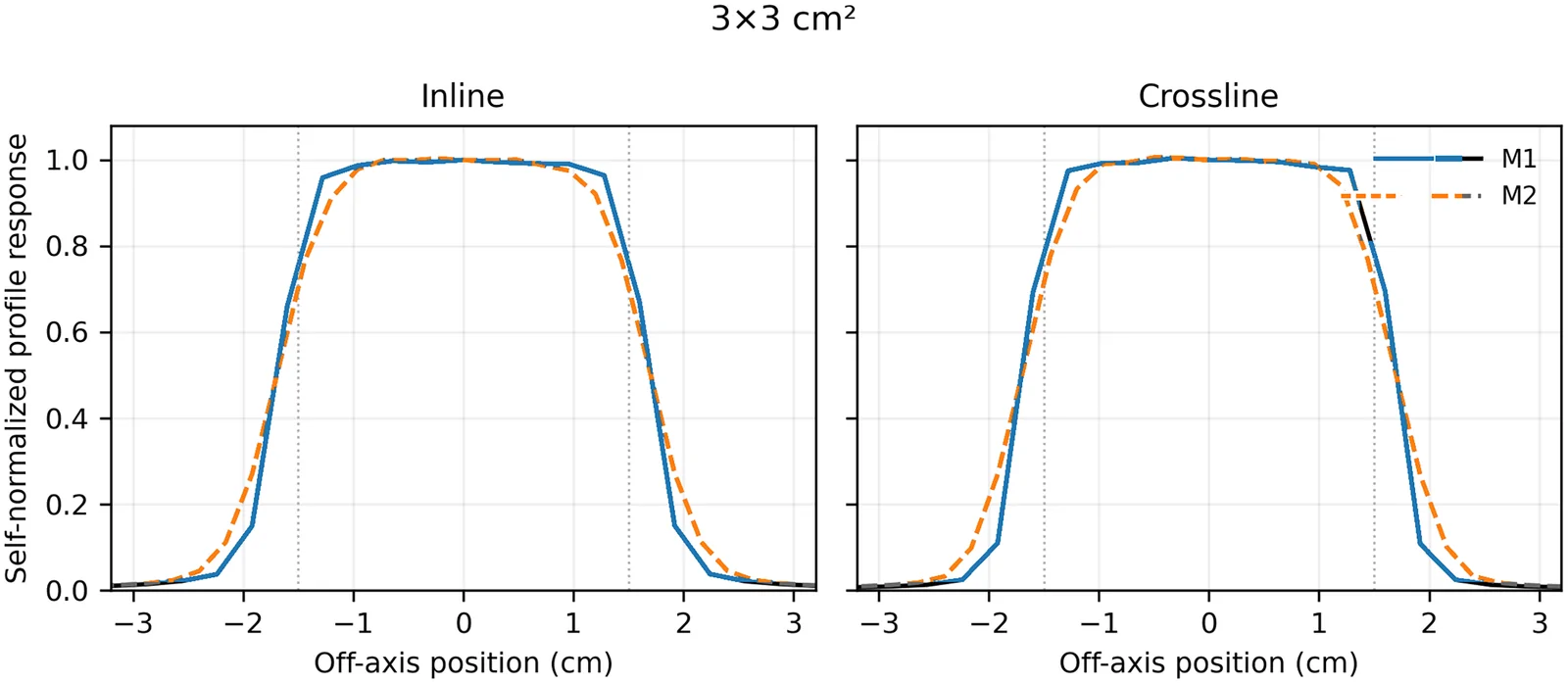
Self-normalized 3 × 3 cm^2^ Monte Carlo profiles for M1 and M2: left panel, inline; right panel, crossline. The light-gray dotted vertical lines indicate the nominal field boundaries.

When both models were referenced to the M1 central-axis value, the field-edge loss showed clear amplification with decreasing field size (**Table 7**).

**Table 7:** Field-edge response using the M1 central-axis value as a common reference.

Field	Axis	M1 − edge	M2 − edge	Difference	M1 + edge	M2 + edge	Difference
0.5×0.5	Inline	0.6854	0.3570	-32.84 pp	0.6957	0.3582	-33.75 pp
0.5×0.5	Crossline	0.6852	0.3605	-32.47 pp	0.6880	0.3608	-32.72 pp
1×1	Inline	0.6879	0.4650	-22.29 pp	0.6937	0.4672	-22.65 pp
1×1	Crossline	0.7157	0.4953	-22.04 pp	0.7143	0.4972	-21.71 pp
2×2	Inline	0.7577	0.6104	-14.73 pp	0.7601	0.6081	-15.20 pp
2×2	Crossline	0.7995	0.5997	-19.98 pp	0.7981	0.5990	-19.91 pp
3×3	Inline	0.7538	0.6436	-11.02 pp	0.7624	0.6451	-11.73 pp
3×3	Crossline	0.7802	0.6529	-12.73 pp	0.7834	0.6493	-13.42 pp

Differences are reported in percentage points (pp).

### 4.5 Angular phase-space sensitivity

The broadened source produced systematic changes in the final pre-phantom angular phase space. **Table 8** summarizes the scalar angular-density moments for all four Monte Carlo field sizes, while **Table 9** and the bin-width analysis compare the corresponding quantile, derived-cone, and tail responses across the same four-field set. The M2 endpoint increased *q*_95_, Ω_95_, and the high-angle tail in every field, whereas individual moment changes were observable-dependent.

**Table 8:** Audited scalar angular-density metrics for the narrow (M1) and broadened (M2) source models.

Field	Model	$\bar{\theta }\left({}^{\circ}\right)$	σ_θ_ (°)	θ_RMS_ (°)	q_95_ (°)	Ω_95_ (sr)	$T_{\theta \geq 1^{\circ }}\left(\%\right)$
0.5×0.5	M1	0.134	0.294	0.323	0.295	8.33×10^−5^	0.439
0.5×0.5	M2	0.258	0.409	0.483	0.578	3.19×10^−4^	0.861
1×1	M1	0.195	0.264	0.328	0.416	1.65×10^−4^	0.378
1×1	M2	0.280	0.336	0.437	0.646	4.00×10^−4^	0.645
2×2	M1	0.356	0.320	0.478	0.714	4.88×10^−4^	0.680
2×2	M2	0.386	0.357	0.525	0.874	7.31×10^−4^	2.260
3×3	M1	0.517	0.401	0.654	1.024	1.00×10^−3^	5.840
3×3	M2	0.539	0.453	0.704	1.156	1.28×10^−3^	10.375

Central estimates are rounded to precision commensurate with the angular-bin sensitivity; history-preserving bootstrap precision was smaller than the displayed angular resolution.

**Table 9:** Field-size dependence of the angular-density quantile response and high-angle-tail sensitivity.

Field	Δ*q*_95_ (°)	*q*_95_ ratio M2/M1	Ω_95_ ratio M2/M1	Tail ratio M2/M1
0.5×0.5	0.283 ± 0.001	1.958 ± 0.002	3.835 ± 0.008	1.961 ± 0.014
1×1	0.230 ± 0.001	1.554 ± 0.001	2.415 ± 0.003	1.708 ± 0.010
2×2	0.160 ± 0.001	1.224 ± 0.001	1.498 ± 0.001	3.324 ± 0.008
3×3	0.132 ± 0.001	1.129 ± 0.001	1.275 ± 0.001	1.776 ± 0.002

Values are direct endpoint comparisons ±1σ history-preserving bootstrap uncertainty.

The field-size amplification is summarized in **Table 9**. The cleanest monotonic angular trend was the M2/M1 ratio of Ω_95_, which increased from 1.275 at 3×3 to 1.498 at 2×2, 2.415 at 1×1, and 3.835 at 0.5×0.5 cm^2^.

History-preserving resampling gave statistical uncertainties much smaller than the observed M1–M2 angular differences. At the nominal 0.05° bin width, ${\text{Ω}}_{95}\left(M2\right)/{\text{Ω}}_{95}\left(M1\right)$ was 3.835 at 0.5×0.5 cm^2^, 2.415 at 1×1 cm^2^, 1.498 at 2×2 cm^2^, and 1.275 at 3×3 cm^2^. The corresponding Δ*q*_95_ values were approximately 0.283°, 0.230°, 0.160°, and 0.132°, respectively, giving the same ordered progression toward the smallest field. The high-angle-tail ratio remained non-monotonic, reinforcing that the magnitude of the endpoint response depends on the observable. Repeating the analysis at 0.025°, 0.05°, and 0.10° preserved the Ω_95_ field-size ordering. For the 2×2 cm^2^ field, the Ω_95_ ratio was 1.498, 1.498, and 1.497 at bin widths of 0.025°, 0.05°, and 010°, respectively; its largest nominal-relative shift was below 0.03%. Across the four-field analysis, the largest tested nominal-relative discretization shift remained approximately 0.91% for the 1×1 cm^2^ field. The discretization dependence was larger than the pure statistical uncertainty in the last displayed digits, so narrative precision was reduced accordingly.

The angular-density profiles shown below illustrate the M1–M2 shape change across all four investigated field sizes using the same BEAMDP-equivalent per-unit-solid-angle definition used for the scalar angular-density analysis. The corresponding *q*_95_ and Ω_95_ metrics are summarized quantitatively in **Table 9** and **Figure 8**. The angular-density profiles are shown in **Figure 9**.

**Figure 8:**
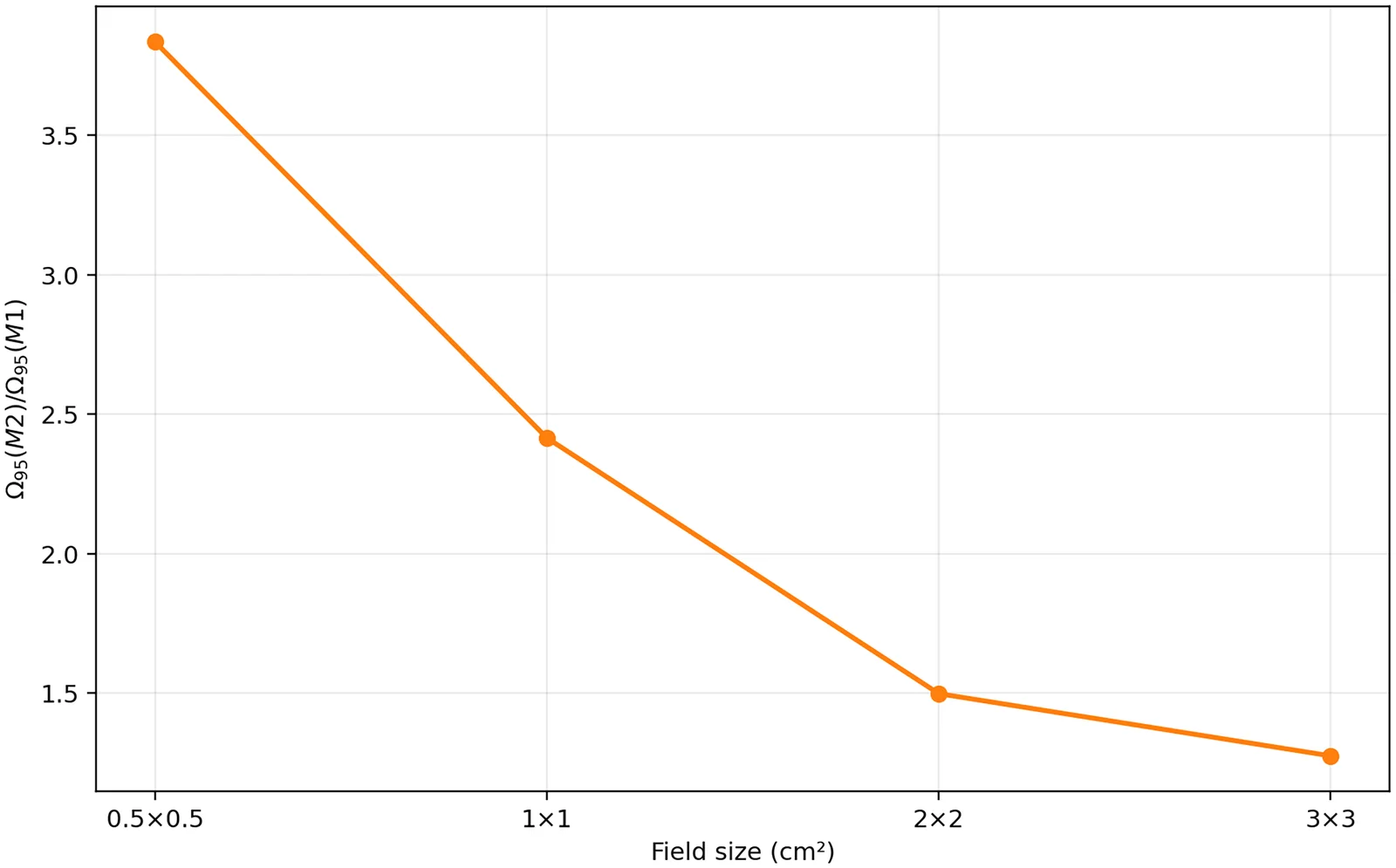
Ratio of the solid angle derived from the 95% angular-density quantile, ${\text{Ω}}_{95}\left(\text{M}2\right)/{\text{Ω}}_{95}\left(\text{M}1\right)$, as a function of field size.

**Figure 9:**
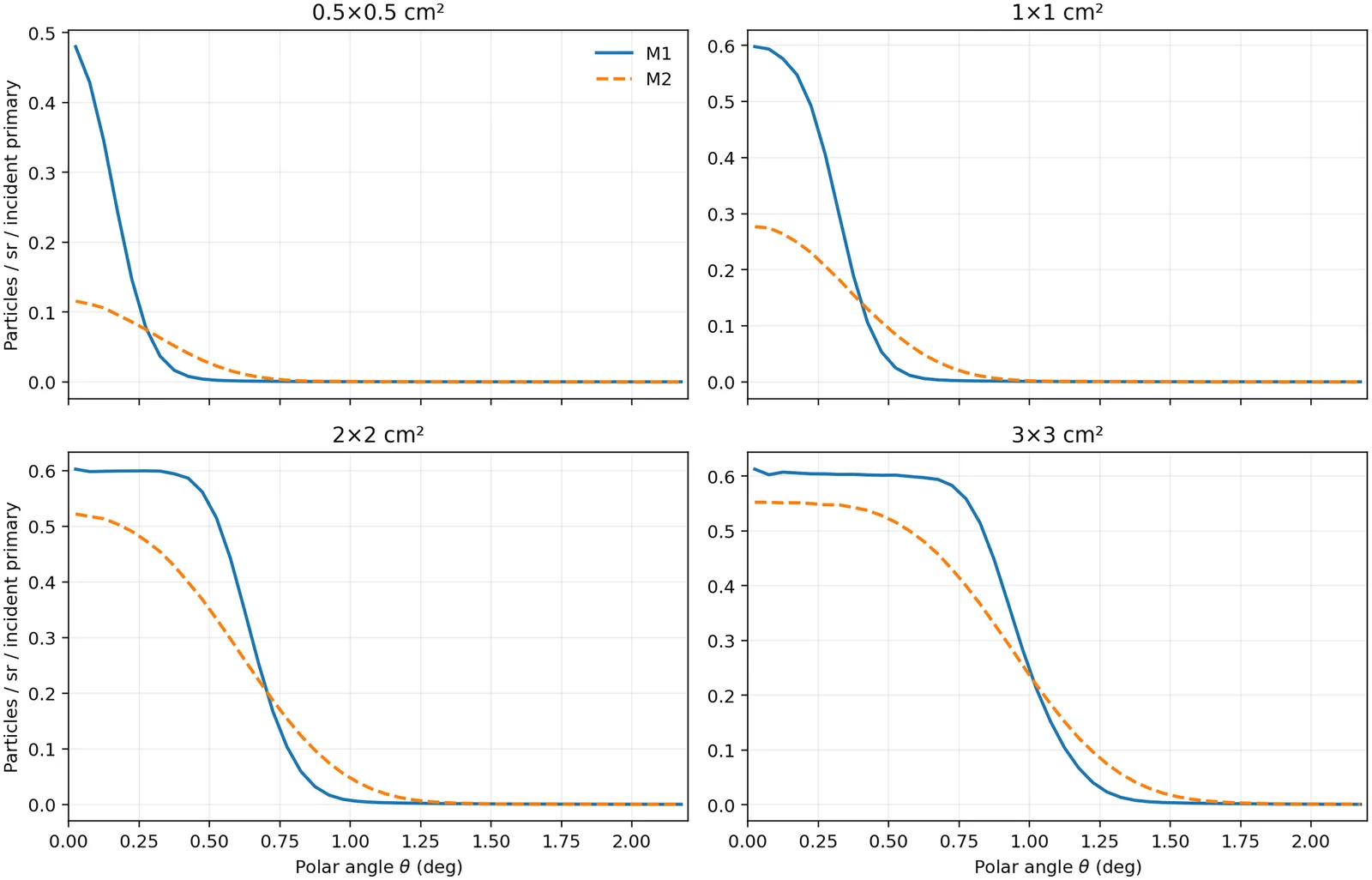
Final pre-phantom photon angular-density profiles for M1 and M2 across the four investigated fields: 0.5×0.5, 1×1, 2×2, and 3×3 cm^2^. The ordinate is particles per unit solid angle per incident primary.

### 4.6 Directional projected-angle response

The projected-angle decomposition showed a larger crossline response in the central photon angular distribution. At *q*_90_, the M2/M1 ratio was 1.051 ± 0.015 inline and 1.619 ± 0.013 crossline for 0.5×0.5 cm^2^, 1.449 ± 0.002 inline and 1.738 ± 0.002 crossline for 1×1 cm^2^, 1.223 ± 0.001 inline and 1.322 ± 0.001 crossline for 2×2 cm^2^, and 1.147 ± 0.001 inline and 1.203 ± 0.001 crossline for 3×3 cm^2^. The projected angular-core FWHM also increased more strongly crossline than inline: 128.5% versus 76.5% at 0.5×0.5 cm^2^, 45.6% versus 22.5% at 1×1 cm^2^, 3.2% versus 1.0% at 2×2 cm^2^, and 0.8% versus 0.4% at 3×3 cm^2^.

In the 0.5×0.5 cm^2^ case, the projected *q*_95_ tails were non-monotonic despite clear $q_{80}/q_{90}$ and core-FWHM broadening: $q_{95,x}$ decreased from about 3.666° to 3.586° and $q_{95,y}$ from about 2.787° to 2.577°. The outermost projected tail is therefore not interpreted as a direct axis-specific source-width surrogate.

### 4.7 Direct comparison at the 3×3 cm^2^ overlap field

At the overlap field, the raw source-model endpoint shift was compared directly with the empirical measurement spread using the same relative-profile measurand (**Table 10**; **Figure 10**). Inline, the source endpoint shift was smaller than the observed measurement spread. Crossline, the source endpoint shift exceeded the observed spread.

**Table 10:** Direct 3×3 cm^2^ comparison of empirical measurement spread and raw source-model endpoint shift.

Position	s_meas_	Δ_source_	${\text{Δ}}_{\text{source}}/s_{\text{meas}}$
Inline +1.5	0.21496	0.05752	0.268
Inline −1.5	0.15159	0.05056	0.334
Crossline +1.5	0.03130	0.07402	2.365
Crossline −1.5	0.04519	0.06682	1.479

**Figure 10:**
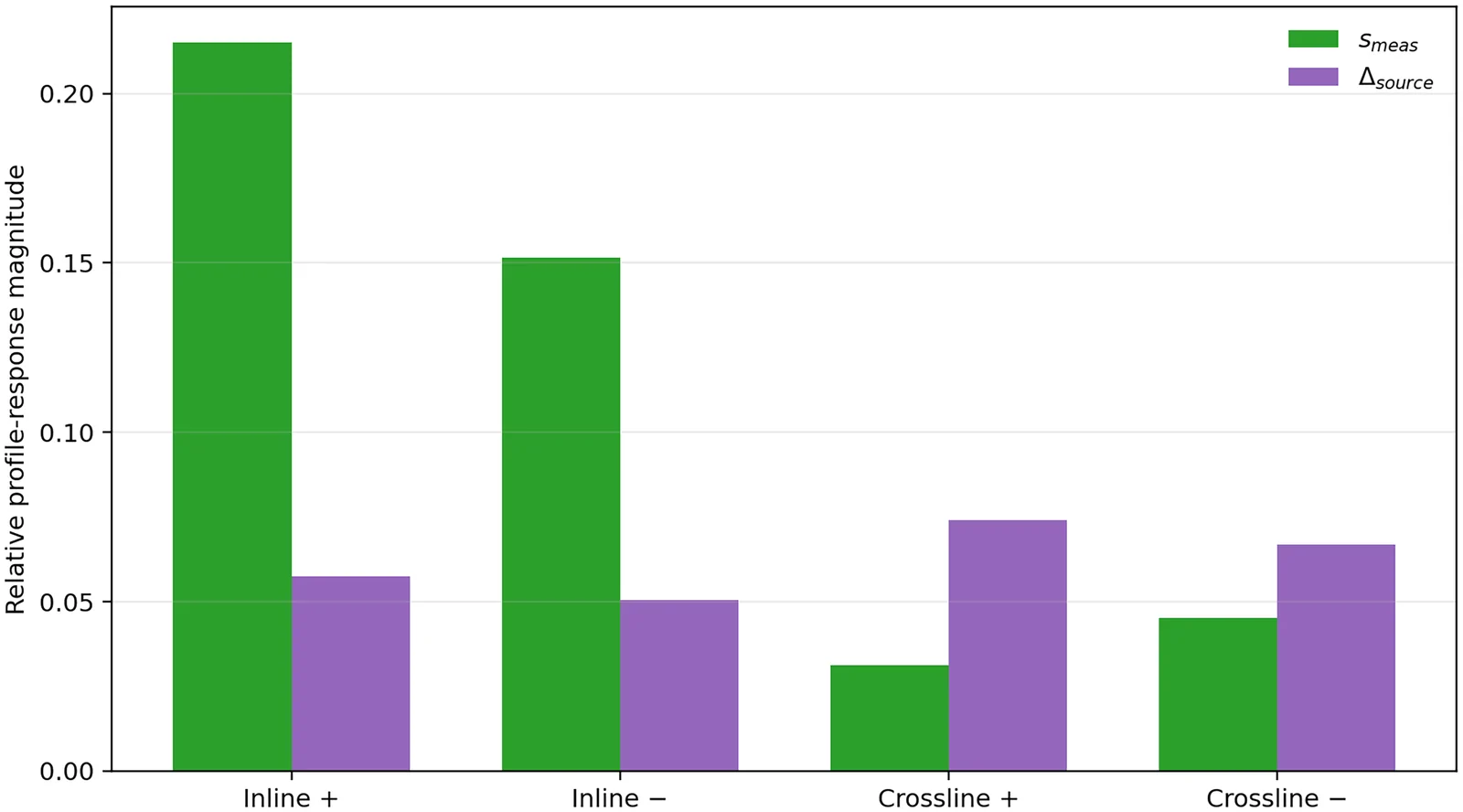
Direct 3×3 cm^2^ comparison between empirical measurement spread and raw source-model profile shift. The source shift is smaller inline but larger crossline.

## 5 Discussion

### 5.1 Main interpretation of the results

The present study shows that the small-field problem contains at least two different components. The first is variability observed during practical measurements. The second is sensitivity of the calculated beam to the effective primary electron source model.

The experimental mean CV increased from 2.46% for the 20×20 cm^2^ field to 15.45% for the 3×3 cm^2^ field. In parallel, the Monte Carlo response to the same prescribed source-model change increased strongly below 3×3 cm^2^. The central-axis reductions were 8.48%, 8.27%, 29.59%, and 56.34% at 3×3, 2×2, 1×1, and 0.5×0.5 cm^2^, respectively. The angular analysis showed similar amplification, with ${\text{Ω}}_{95}^{M2}/{\text{Ω}}_{95}^{M1}$ increasing from 1.275 to 1.498, 2.415, and 3.835.

These results indicate a change in model sensitivity as the field becomes smaller. At 3×3 cm^2^, source-width effects are already visible within the field that also has experimental measurements. At 2×2, 1×1 and 0.5×0.5 cm^2^, the responses are Monte Carlo endpoint-sensitivity results only and were not experimentally validated in the present study. The supported conclusion is therefore that the calculated downstream response becomes increasingly sensitive to the prescribed effective source state as the aperture is reduced.

### 5.2 Measurement variability in small fields

The increase in empirical measurement variability with decreasing field size agrees with the known difficulty of small-field dosimetry (1, 3, 4). Small fields combine steep dose gradients, detector-volume effects, positioning sensitivity, loss of lateral charged-particle equilibrium, detector perturbation, and field-size-dependent detector response.

The present experimental analysis was not designed as a conventional detector-characterization study. Five detectors with different geometries were used, and the measurements were obtained during different periods. The observed spread can therefore contain detector response, positioning and setup, environmental conditions, temporal beam variation, and operator-related effects. This is why it is described as empirical measurement variability rather than pure detector uncertainty.

The 3×3 cm^2^ field showed a strong directional difference: inline edge CV values were approximately 25–26%, whereas crossline values were approximately 5–6%. The present measurement dataset alone does not identify one unique physical cause for this difference. Axis-dependent detector response, positioning, collimation, or accelerator-state effects could contribute, but the available data do not separate these possibilities. The directional difference is therefore treated as an observed property of this dataset and is not attributed specifically to the primary source model.

When experimental variability itself becomes large, disagreement between calculation and measurement is more difficult to assign to one physical source. This supports recent work showing that the small-field problem cannot be reduced to detector performance alone (2, 3, 5).

The normalized-position analysis qualifies the aggregate field-size trend. The aggregate mean CV increases as field size decreases, but those field means contain different mixtures of interior and boundary positions. At the common boundary ξ=1, the inline variability increases monotonically from 5.33% to 25.54%, whereas the crossline response is smaller and non-monotonic. Accordingly, the experimental result is interpreted as a field-size-, position-, and axis-dependent empirical variability pattern rather than a single detector-independent field-size law. Previously reported 1×1 and 0.5×0.5 cm^2^ measurements were output-factor measurements with Razor diode and CC04 detectors (8), not a matched five-detector lateral-profile dataset at the normalized positions analysed here; the present sub-centimeter profile results therefore remain Monte Carlo endpoint-sensitivity results only.

### 5.3 Increasing sensitivity to primary electron source width

The central-axis response showed strong amplification in the sub-centimeter regime, but the four-field CAX response is not strictly monotonic because the reductions at 3×3 and 2×2 cm^2^ are nearly equal. The same transition from M1 to M2 was used at every field. Thus, the increasing response was not produced by applying a larger perturbation to the smaller fields. Instead, the same source-model change produced a larger downstream consequence. This is the meaning of field-size amplification in the present study.

Previous work supports the importance of source representation in small fields. Borzov et al. showed that reconstructed incident electron-source distribution can affect small-field output factors when source occlusion becomes important (6). Fogliata et al. showed that effective spot-size parameters influence output factor and penumbra for small MLC fields (7). Our previous work also demonstrated dosimetric sensitivity to primary electron source width (8). The present analysis extends these observations by examining four Monte Carlo field sizes together with the associated angular phase-space response. The cleanest four-field progression is observed in Ω_95_; CAX, Δ*q*_95_, penumbra, and high-angle-tail responses show that amplification is observable-dependent.

The common-reference edge response showed the same general direction: source-related edge reductions were approximately 11–13 percentage points at 3×3 cm^2^, 15–20 percentage points at 2×2 cm^2^, approximately 22 percentage points at 1×1 cm^2^, and 32–34 percentage points at 0.5×0.5 cm^2^. This indicates that the effective source representation becomes increasingly important when the aperture accepts a smaller part of the available radiation field, while retaining axis- and observable-dependent detail.

### 5.4 Directional projected-angle response and scope

The projected-angle reanalysis resolves the directional information that is lost when the anisotropic M2 perturbation is reduced to a scalar polar angle. The larger crossline response in $q_{80}/q_{90}$ and in the angular-core FWHM is qualitatively consistent with the larger prescribed crossline broadening in M2. However, M1-to-M2 changes both transverse source widths simultaneously, so the directional decomposition does not isolate independent *x*-only, *y*-only, or interaction effects. Scalar *q*_95_ and Ω_95_ are retained as measures of the normalized polar angular-density redistribution and are not used as direct evidence for axis-specific crossline broadening. Likewise, ${\text{Δ}}_{\text{source}}/s_{\text{meas}}$ is retained only as a descriptive overlap-field comparison, not as an axis-specific physical consistency test.

### 5.5 Penumbra and the spatial expression of source broadening

The broadened source increased the 20–80% penumbra in every evaluated field and direction. This behaviour is physically reasonable because a broader effective source produces a larger range of ray origins and therefore a more gradual geometrical field edge after collimation. The observation is consistent with the known relationship between effective source size and penumbra (7, 8).

The relative penumbra increase did not follow a simple monotonic field-size sequence. The largest percentage increase occurred for the 1×1 cm^2^ crossline profile rather than the smallest field. This illustrates why no single profile quantity should be used alone to describe source sensitivity. Central-axis response, edge response, penumbra, and angular phase space contain complementary information.

### 5.6 Why self-normalized profiles can hide part of the source effect

The self-normalized profile results behaved differently from the common-scale response. In the 0.5×0.5 cm^2^ field, the normalized M2 edge response became higher than the corresponding M1 value even though the absolute M2 response was much lower. This is caused by the very strong reduction of the M2 central-axis value.

Thus, normalized profile shape and overall profile-response amplitude are not the same physical quantity. A profile can appear similar, or even show an apparently larger edge-to-center ratio, after independent normalization while a substantial absolute-response change has been removed by the normalization. This may be important during beam modelling and commissioning, and it motivated the use of both self-normalized and common-reference comparisons in the present study.

### 5.7 Angular phase space as an intermediate physical level

The angular analysis provides a physical connection between source representation and dose response. The broadened source changed not only the dose profiles but also the final pre-phantom photon angular distribution. The cleanest monotonic angular result was the Ω_95_ ratio, which increased from 1.275 at 3×3 to 1.498 at 2×2, 2.415 at 1×1, and 3.835 at 0.5×0.5 cm^2^.

This indicates that the source-width perturbation remains expressed in the angular structure of the photon field after transport through the treatment head. The interpretation is consistent with Martins et al., who showed that directional information in a clinical phase-space file affects calculated dose profiles (16). It is also conceptually consistent with the Monaco virtual-source architecture, where source fluence, energy, spatial size, and angular characteristics are included in the source representation (10).

The scalar polar-angle *q*_95_ and Ω_95_ metrics are retained as global measures of normalized angular-density redistribution. Directional interpretation is based separately on the projected *θ_x_* and *θ_y_* analysis; the scalar containment metrics are not used as direct evidence for a larger response on one axis.

The present study differs because the angular distribution is treated as an observable itself rather than only as an input to a downstream dose engine. This allows the sequence illustrated in Equation 42:

(42) primary electron source width→photon angular phase space→dose response

to be examined at two downstream levels.

### 5.8 Very small fields as restrictive phase-space filters

One possible interpretation of the observed amplification is that a very small aperture acts as a restrictive phase-space filter. A larger field accepts a wider range of particle positions and directions, so a source-state perturbation can be averaged over a relatively large accepted phase space. When the aperture becomes very small, only a restricted part of the available spatial and angular phase space contributes to the transmitted field. Under these conditions, the same change in the source distribution can produce a larger relative change in the transmitted beam.

In this limited sense, very small fields may retain stronger source-state memory than larger fields. The term does not describe a new fundamental memory mechanism. It refers only to the observation that a restricted field can preserve greater sensitivity to differences in the effective source representation. The strong CAX and Ω_95_ trends support this interpretation, but the non-monotonic self-normalized profile behaviour shows that it is not a universal scaling law for every observable.

### 5.9 Measurement variability versus source-model sensitivity at 3×3 cm^2^

The 3×3 cm^2^ field was the only field where experimental and Monte Carlo quantities could be directly compared using the same center-normalized profile measurand. In this specific dataset, the raw source endpoint shift was approximately 27–33% of the experimental spread inline and approximately 1.48–2.37 times the observed spread crossline. These directional ratios are descriptive of the present overlap field and are not generalized as universal axis-dependent source sensitivities.

This comparison is descriptive: *s*_meas_ is the empirical inter-detector/session spread and Δ_source_ is the raw M1–M2 endpoint shift. No probability distribution, independence assumption, or combined variance budget is assigned to these two quantities in the revised analysis.

### 5.10 Implications for clinical beam modelling

The present results are relevant to the structure of modern treatment-planning algorithms. Acuros XB begins from an effective photon beam source model before solving downstream transport (9). Monaco uses a virtual-source representation containing source-size, fluence, spectrum, and angular information before XVMC dose transport (10). The transport methods are different, but both depend on an effective source representation.

Improving downstream transport accuracy therefore does not automatically remove sensitivity to source modelling. The present Monte Carlo endpoint analysis shows increasing source-model sensitivity as the simulated field is reduced. This does not establish machine-specific sub-centimeter clinical behaviour, because the 1×1 and 0.5×0.5 cm^2^ cases were not experimentally validated here. Rather, the result motivates future commissioning studies and independent Monte Carlo validation work to include explicit source-parameter sensitivity testing when extending models into the sub-centimeter regime (3, 5, 7).

### 5.11 Prospective dynamic-source interpretation and upstream–downstream consistency

The present numerical evidence is stationary: it demonstrates the chain summarized in Equation 43:

(43) effective primary-electron source state→linac-head transport→photon phase space→dose/profile response.

The broader inference is that a fitted or prescribed stationary source width is a reduced representation and need not correspond uniquely to one instantaneous physical source state. By the total-covariance decomposition, the same effective covariance can in principle contain both within-state particle spread and between-state centroid variability. The present calculations do not identify that decomposition; they establish the downstream sensitivity to the effective source representation.

The literature-derived *c_T_* provides a physically interpretable, but deliberately limited, bridge to accelerator steering. Its dimensions are those of a response coefficient (mm/mA), and the two Chojnowski reconstructions agree to about 2.35% within the same published dataset. This internal agreement supports the use of $\left|c_{T}\right|\approx 0.020$ mm/mA as a local inferential response scale for hypothesis construction. It does not establish a universal calibration or show that M2 arose from 2T motion. The independent van Appeldoorn current-to-tilt result supports the same order of steering-current sensitivity at the beam-response level without constituting an independent focal-spot coefficient.

A decisive future test follows directly. A genuinely state-conditioned or time-sliced source ensemble can be transported through the same treatment head and compared with a stationary Gaussian matched to the same Σ_eff_. If the resulting photon phase space and dose agree within relevant uncertainty, the stationary covariance is an adequate reduced representation in that regime. If they differ despite matched effective covariance, the temporal/state structure contains transport-relevant information that is lost by stationary reduction.

This question also has a more specific physical motivation in the context of emerging time-aware delivery. DMAT explicitly incorporates finite acceleration, deceleration, axis synchronization, and evolving gantry/MLC/dose-rate states into the downstream delivery description (17). Independently, gantry-angle-dependent electron-beam steering is already an established feature of Elekta linac control: the applied steering current contains a gantry-dependent lookup-table contribution together with servo correction, and measured 2R/2T service-graph information can be used to optimize beam control during rotation (19). These two observations do not demonstrate that gantry acceleration itself causes temporal primary-electron source fluctuations. They do, however, identify a physically plausible control pathway that can now be tested: rapidly evolving mechanical and control states may couple to steering current and therefore to the effective electron-source state. A future model can express this without assuming statistical independence as p(r,p,E∣q(t)), where *q*(*t*) may include measured steering-current and delivery-state variables such as gantry angle and, when experimentally available, angular velocity or acceleration. In that formulation the prescribed delivery variables and the upstream source state are conceptually distinguishable but potentially coupled. The present stationary M1–M2 calculations provide the endpoint-sensitivity baseline required to determine whether such coupling becomes transport-relevant; they do not establish that the coupling occurs or that it explains M2.

### 5.12 Limitations

Several limitations should be considered. First, the experimental measurements and Monte Carlo calculations were not a machine-specific digital-twin comparison. Measurements were performed on a clinical Elekta Versa HD, whereas the simulation used a generalized Versa HD/Agility model based on published information and previous modelling work. The simulated source states should not be interpreted as measurements of the actual source state of that individual accelerator.

Second, empirical measurement variability was obtained from five detector datasets acquired during different measurement periods. This captures practical combined spread but does not allow detector response, positioning, environment, temporal machine variation, and operator effects to be separated.

Third, direct experimental-versus-source comparison was possible only at 3×3 cm^2^. The 2×2, 1×1 and 0.5×0.5 cm^2^ findings are Monte Carlo endpoint-sensitivity results only and should not be interpreted as experimentally validated clinical behaviour.

Fourth, only two primary electron source states were investigated. The study therefore quantifies an endpoint sensitivity rather than the complete functional dependence of dose or angular phase space on source width. Fifth, the angular distributions were obtained after target/head transport at the final pre-phantom scoring plane and are not isolated target-emission distributions.

Sixth, the directional projected-angle analysis resolves inline and crossline response of the prescribed M1-to-M2 endpoint change, but it does not isolate independent *x*-only and *y*-only source perturbations because both source dimensions change simultaneously. Seventh, the history-preserving resampling estimates finite-history precision conditional on each archived phase-space realization. Each field/source-model combination was generated in a separate BEAMnrc run and a separate phase-space file, as described in the Methods. Angular-bin-width sensitivity was evaluated explicitly and the principal field-size ordering was unchanged. Finally, the prospective dynamic-source interpretation remains theoretical: no time-resolved source dynamics were measured or simulated, and no unique accelerator-control-to-source mapping is claimed.

In addition, the M1 benchmark in the present manuscript is quantitative for depth-dose behaviour but does not provide an independent absolute lateral-profile benchmark. The lateral-profile results are therefore interpreted as a controlled numerical sensitivity analysis of the prescribed M1-to-M2 endpoints, not as machine-specific predictions of absolute clinical lateral beam characteristics.

### 5.13 Overall interpretation

Taken together, the results support a simple physical picture. As field size decreases, practical measurement variability increases, while the calculated beam simultaneously becomes more sensitive to the effective primary electron source representation. The source-model effect is visible in central-axis response, common-reference edge response, penumbra, and photon angular phase space. Across the four Monte Carlo fields, the cleanest monotonic trend is the solid angle derived from the 95% angular-density quantile; central-axis response is strongly amplified only below 2×2 cm^2^ and is not strictly monotonic across all four fields.

The main result is therefore not that source modelling replaces measurement-related challenges in very small fields. Rather, source modelling represents an additional sensitivity channel whose calculated effect becomes more pronounced as the field is reduced.

This relationship is summarized in Equation 44:

(44) smaller field→more restricted accepted phase space→greater sensitivity to the effective source state.

The study connects primary electron source representation, photon angular phase space, and small-field dosimetric response, and suggests that very small fields provide a sensitive test of how well an effective source representation describes the radiation beam used by dose-calculation models.

## 6 Conclusion

The present 6 MV study demonstrates a stationary endpoint-sensitivity chain linking effective primary-electron source representation to linac-head transport, final pre-phantom photon angular phase space, and small-field dose/profile response. Across the four Monte Carlo fields, the Ω_95_ M2/M1 ratio progresses from 1.275 at 3×3 to 1.498 at 2×2, 2.415 at 1×1, and 3.835 at 0.5×0.5 cm^2^, whereas the CAX reduction remains nearly unchanged from 3×3 (8.48%) to 2×2 (8.27%) before increasing strongly at 1 cm and sub-centimeter fields. Field-size amplification is therefore real within the investigated endpoint comparison but is observable-dependent rather than a universal monotonic law.

The present evidence does not demonstrate a dynamic source. Instead, it shows why the adequacy of a stationary effective source becomes an important modelling question when the accepted phase space is highly restricted. The reduced covariance relation provides a precise prospective interpretation: a fluence-integrated stationary source may contain both intrinsic within-state spread and unresolved centroid-state variability. The literature-derived transverse steering-to-focal-spot coefficient $\left|c_{T}\right|\approx 0.020$ mm/mA is retained as a physically interpretable local response scale for future hypothesis testing, with its scope explicitly limited to a figure-derived local coefficient rather than a manufacturer or machine-specific calibration.

The next decisive step is to compare a genuinely state-conditioned or time-sliced source calculation with a stationary source matched to the same effective covariance. Agreement would identify a regime in which the stationary source is an adequate reduced model; disagreement would show that source-state history carries transport-relevant information not preserved by stationary reduction. In this sense, the current work establishes the stationary numerical foundation for a future dynamic-source test without presenting that future model as an accomplished simulation result.

## Data Availability

For reproducibility, the complete BEAMnrc input files used for the source models and simulated field configurations are archived on Zenodo at https://doi.org/10.5281/zenodo.22694517. The exact archived inputs provide the full simulation configuration, including the field-specific jaw/MLC aperture definitions, without reproducing every leaf and jaw setting in the manuscript text. The datasets generated and/or analysed during the current study remain available from the corresponding author on reasonable request.

## Appendix

### Appendix A Symbols, physical quantities, and units

Table A1 defines the principal symbols, physical quantities, and units used in the theoretical and numerical analysis, including the prospective effective-source quantities and the literature-derived local response coefficient *c_T_*.

**Table A1:** Symbols, physical quantities, and units used in this article.

Symbol	Physical meaning	Unit/dimension	Notes
M1, M2	Prescribed narrow and broadened stationary primary-electron source endpoints	–	Not measured confidence bounds
*x*, *y*	Inline and crossline transverse coordinates	cm or mm	Source/profile convention
FWHM*_x_*, FWHM*_y_*	Gaussian source full widths at half maximum	cm	FWHM≃2.355σ
*D_M_*(*x*)	Monte Carlo profile dose ordinate for source model *M*	dose per primary history	Common DOSXYZnrc normalization
*R*_CAX_	M2/M1 central-axis dose ratio	dimensionless	$D_{M2}\left(0\right)/D_{M1}\left(0\right)$
*P*_20–80_	20–80% penumbra width	cm	Crossing positions linearly interpolated
*W*_50_	50% profile width	cm	Crossing positions linearly interpolated
*θ*	Photon polar angle at final pre-phantom phase-space plane	degree or rad	Scalar angular analysis
*W_i_*	Summed photon statistical weight in polar-angle bin *i*	statistical weight	Raw phase-space bin weight
*N*_inc_	Incident-primary count stored in the phase-space header	histories	Angular-density normalization
ΔΩ*_i_*	Annular solid angle of polar-angle bin *i*	sr	$2\pi \left(\text{cos}\theta_{\text{low}}-\text{cos}\theta_{\text{high}}\right)$
*y_i_*	Angular-density ordinate $W_{i}/\left(N_{\text{inc}}{\text{ΔΩ}}_{i}\right)$	incident-primary^−1^ sr^−1^	Applied consistently across all four fields
*p_i_*	Normalized angular-density weight in polar-angle bin *i*	dimensionless	$y_{i}\text{Δ}\theta_{i}/\sum_{j}y_{j}\text{Δ}\theta_{j}$
*θ_x_*, *θ_y_*	Projected inline/crossline photon angles	degree or rad	From phase-space direction cosines
*q*_95_	95% quantile of the normalized polar angular-density profile	degree or rad	From cumulative *p_i_*
Ω_95_	Solid angle of the cone defined by angular-density *q*_95_	sr	Derived angular-density spread metric
*p_n_*(*r*)	Normalized instantaneous source-state distribution	area^−1^	Prospective only
*w_n_*	Fluence fraction of state *n*	dimensionless	$\sum_{n}w_{n}=1$
*p*_eff_(*r*)	Fluence-integrated effective source distribution	area^−1^	$\sum_{n}w_{n}p_{n}$
Σ_inst_	Intrinsic within-state source covariance	length^2^	Prospective decomposition
Σ_eff_	Covariance of effective stationary source	length^2^	Total covariance
*C_μ_*	Covariance of source-state centroids	length^2^	Between-state contribution
*X_s_*	One-dimensional instantaneous source-centroid coordinate	cm or mm	Special case
σ_0_	Common intrinsic source standard deviation	cm or mm	Special case
*c_T_*	Transverse steering-to-focal-spot position response coefficient $\partial x_{f}/\partial I_{2T}$	mm/mA	Literature-derived local slope
*x_f_*	Transverse focal-spot position	mm	Used in *c_T_* definition
*I*_2_ *_T_*	Transverse 2T steering current	mA	No time series measured here
*q*(*t*)	Generic evolving accelerator state	model-dependent	Prospective state-conditioned source notation

## References

[1] Palmans H Andreo P Huq MS Seuntjens J Christaki KE Meghzifene A . Dosimetry of small static fields used in external photon beam radiotherapy: Summary of TRS-483, the IAEA–AAPM international code of practice for reference and relative dose determination. Med Phys. (2018) 45:e1123–45. doi: 10.1002/mp.13208 30247757

[2] Decabooter E Essers M Gershkevitsh E Hussein M Kry SF Lehmann J . Patient specific quality assurance: The final safety net in radiotherapy. Radiotherapy Oncol. (2026) 221:111597. doi: 10.1016/j.radonc.2026.111597 42134659

[3] Nishioka K Kunii Y Tanabe Y Sakamoto Y Nakamoto A Takahashi S . Evaluation of the small-field output factor in Eclipse modeling methods using representative beam and measured data with averaged ionization chamber and diode detector measurements. Phys Eng Sci Med. (2026) 49:417–32. doi: 10.1007/s13246-025-01676-1 41284147 PMC12987896

[4] Lechner W Palmans H . Uncertainty estimation for dosimetry in radiation oncology. Phys Imaging Radiat Oncol. (2025) 34:100773. doi: 10.1016/j.phro.2025.100773 40521102 PMC12164212

[5] Fogliata A Stravato A Dusi F Menna S Pilloni E Quaranta FV . Small field dosimetry on a ring-shape Linac machine with a dual-layer MLC. Med Phys. (2026) 53:e70310. doi: 10.1002/mp.70310 41615055

[6] Borzov E Nevelsky A Bar-Deroma R Orion I . Reconstruction of the electron source intensity distribution of a clinical linear accelerator using in-air measurements and a genetic algorithm. Phys Imaging Radiat Oncol. (2019) 12:67–73. doi: 10.1016/j.phro.2019.11.010 33458298 PMC7807614

[7] Fogliata A Stravato A Pelizzoli M La Fauci F Gallo P Bresolin A . Effective spot size parameters for Acuros dose calculation algorithm using enhanced leaf modelling: Estimation based on small rectangular MLC fields. J Appl Clin Med Phys. (2025) 26:e70315. doi: 10.1002/acm2.70315 41201191 PMC12593538

[8] Habib MM Abdelgawad MH Guirguis A Alharbi M El-Maraghy K Sallam AM . Dosimetric investigation of small fields in radiotherapy measurements using Monte Carlo simulations, CC04 ionization chamber, and razor diode. Phys Eng Sci Med. (2025) 48:813–25. doi: 10.1007/s13246-025-01546-w 40522570 PMC12208963

[9] Varian Medical Systems . Eclipse Photon and Electron Algorithms Reference Guide (Palo Alto, California: Varian Medical Systems, Inc.). (2015), Document P1008611-003-C, March 2015. 2015.

[10] Elekta AB . Monaco XVMC and Pencil Beam Dose Calculation Reference Manual. Stockholm: Elekta AB. (2024), Document E014439/02, Virtual Source Specific Models (VSM 1.6). 2024.

[11] Chen ZH Riis HL White R Milan T Rowshanfarzad P . A comprehensive investigation of the radiation isocentre spatial variability in linear accelerators: implications for commissioning, QA, and clinical protocols. Phys Eng Sci Med. (2025) 48:1979–93. doi: 10.1007/s13246-025-01637-8 40928608 PMC12738597

[12] Chojnowski JM Sykes JR Thwaites DI . Practical beam steering of X-ray beams on Elekta accelerators: The effect of focal spot alignment on beam (symmetry and position) and radiation isocentre (size and position). Phys Eng Sci Med. (2020) 43:1441–50. doi: 10.1007/s13246-020-00926-8 32997299

[13] Riis HL Ebert MA Rowshanfarzad P . Detection of the focal spot motion relative to the collimator axis of a linear accelerator under gantry rotation. Phys Med Biol. (2019) 64:03NT02. doi: 10.1088/1361-6560/aafa04 30566917

[14] St-Aubin J Steciw S Kirkby C Fallone BG . An integrated 6 MV linear accelerator model from electron gun to dose in a water tank. Med Phys. (2010) 37:2279–88. doi: 10.1118/1.3397455 20527562

[15] St-Aubin J Steciw S Fallone BG . Effect of transverse magnetic fields on a simulated in-line 6 MV linac. Phys Med Biol. (2010) 55:4861–9. doi: 10.1088/0031-9155/55/16/015 20679699

[16] Martins JC Saxena R Neppl S Alhazmi A Reiner M Veloza S . Optimization of Phase Space files from clinical linear accelerators. Physica Med. (2019) 64:54–68. doi: 10.1016/j.ejmp.2019.06.007 31515036

[17] Li T Kuusela E Ruokokoski E Hyvönen H Jaboin J Myllykoski M . Dynamic modulated arc therapy (DMAT): A time-aware, modulation-steered optimization framework for next-generation radiotherapy delivery. J Appl Clin Med Phys. (2026) 27:e70764. doi: 10.1002/acm2.70764 42638435 PMC13504227

[18] van Appeldoorn AA Kok JGM Wolthaus JWH . Gantry angle dependent beam control optimization of a traveling wave linear accelerator to improve VMAT delivery. J Appl Clin Med Phys. (2020) 21:312–21. doi: 10.1002/acm2.13047 33094890 PMC7700923

[19] van Appeldoorn AA Kok JGM Wolthaus JWH . New gantry angle-dependent beam control optimization with Elekta linear accelerator for VMAT delivery. J Appl Clin Med Phys. (2025) 26:e14598. doi: 10.1002/acm2.14598 39776233 PMC11905234

[20] Minty MG Zimmermann F . Orbit measurement and correction. In: Measurement and Control of Charged Particle Beams. Particle Acceleration and Detection. Springer, Berlin, Heidelberg (2003). p. 69–98. doi: 10.1007/978-3-662-08581-3_3

[21] Chand B Singh R Kumar M . Determination and validation of the initial beam parameters of Elekta Agility collimator head by Monte Carlo simulations. Phys Eng Sci Med. (2022) 45:889–99. doi: 10.1007/s13246-022-01159-7 35849322

[22] Gholampourkashi S Cygler JE Belec J Vujicic M Heath E . Monte Carlo and analytic modeling of an Elekta Infinity linac with Agility MLC: Investigating the significance of accurate model parameters for small radiation fields. J Appl Clin Med Phys. (2019) 20:55–67. doi: 10.1002/acm2.12485 30408308 PMC6333188

[23] Kawrakow I Mainegra-Hing E Rogers DWO Tessier F Walters BRB . The Egsnrc Code System: Monte Carlo Simulation of Electron and Photon Transport. Ottawa: National Research Council Canada. (2025), NRCC Report PIRS-701. National Research Council Canada. 2025.

[24] Rogers DWO Faddegon BA Ding GX Ma C-M Wei J Mackie TR . BEAM: A Monte Carlo code to simulate radiotherapy treatment units. Med Phys. (1995) 22:503–24. doi: 10.1118/1.597552 7643786

[25] Rogers DWO Walters BRB Kawrakow I . Beamnrc Users Manual. Ottawa: National Research Council Canada. (2025), NRCC Report PIRS-509a. National Research Council Canada. 2025.

[26] Walters BRB Kawrakow I Rogers DWO . Dosxyznrc Users Manual. Ottawa: National Research Council Canada. (2025), NRCC Report PIRS-794. National Research Council Canada. 2025.

[27] Ma CM, Rogers DWO . BEAMDP Users Manual. NRCC Report PIRS-0509(C), rev A. Ottawa: National Research Council Canada. (2004).

[28] Ajaz M Alrebdi HI Younis H Al-mugren KS . Environmental absorbed-dose quantities for natural radionuclides in soil using a simplified block phantom with an ICRP 110 comparison. J Environ Radioact. (2026) 299:108122. doi: 10.1016/j.jenvrad.2026.108122 42551211

[29] Walters BRB Kawrakow I Rogers DWO . History by history statistical estimators in the BEAM code system. Med Phys. (2002) 29:2745–52. doi: 10.1118/1.1517611 12512706

