# Supplementary material for: Toward dynamic Monte Carlo source modeling: measurement variability and primary electron source-width sensitivity in very small photon fields
Source: Front Oncol. 2026 Oct 28;16:1968378. doi: 10.3389/fonc.2026.1968378 (PMC13751441; doi:10.3389/fonc.2026.1968378)
Supplement: Supplementary file 1 [file Supplementaryfile1.pdf]

10.3389/fonc.2026.1968378

Supplementary Material

Toward Dynamic Monte Carlo Source Modeling: Measurement Variability and Primary Electron Source-Width Sensitivity in Very Small Photon Fields

Mina M. Habib

Supplementary Figure S1

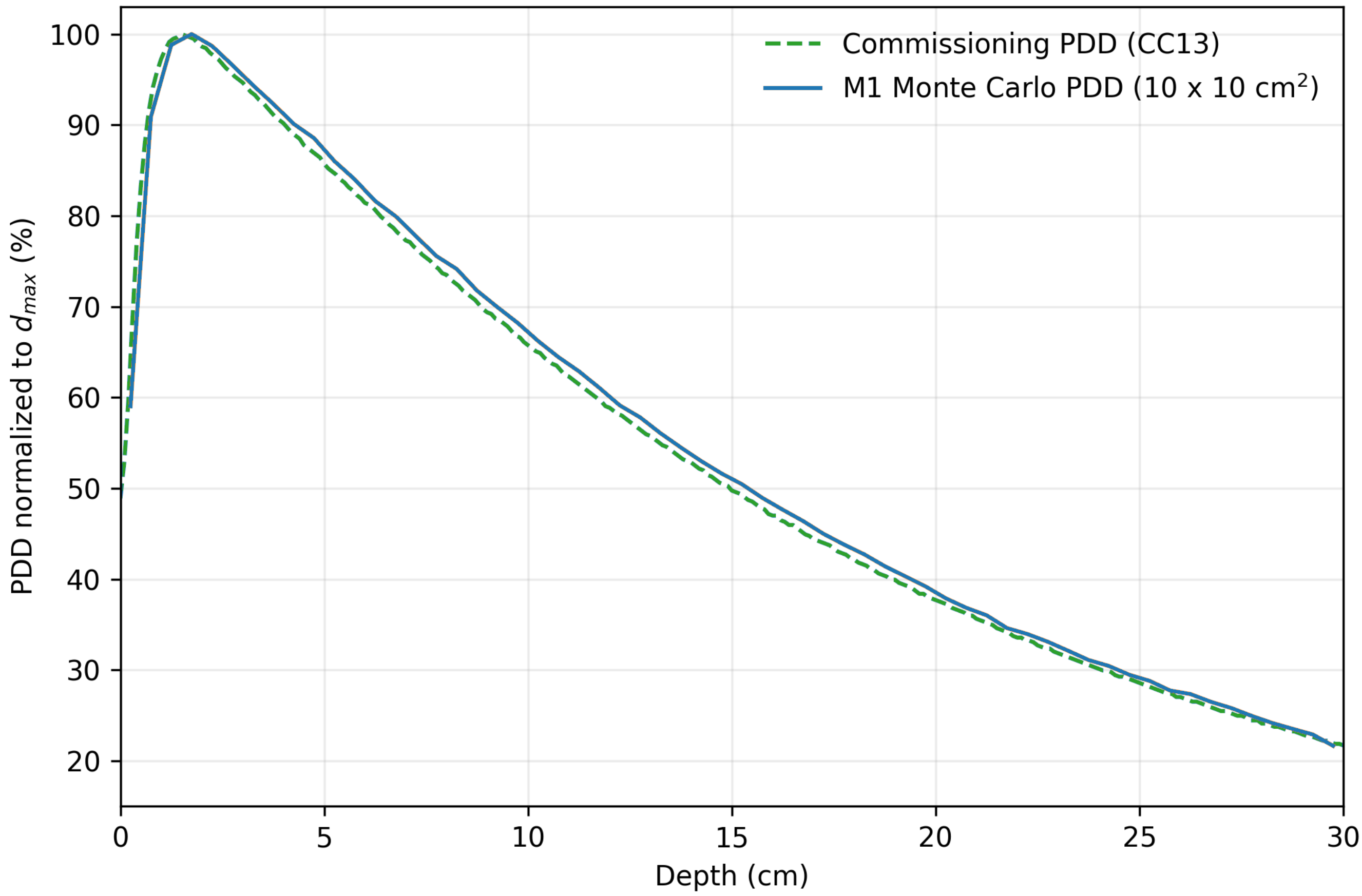

Supplementary Figure S1. M1 Monte Carlo 10 × 10 cm^2 PDD compared with the CC13 commissioning PDD after independent normalization to each curve's d_{max}. Quantitative agreement for z ≥ 2 cm: mean absolute difference 0.97 percentage point, RMS difference 1.04 percentage points, maximum absolute difference 1.68 percentage points, with all 56 evaluated Monte Carlo depth points within ±2 percentage points.

Supplementary Tables

Supplementary Table S1. Matched normalized-position comparison at the common field boundary, $\xi = 1$.

Field	Aggregate mean CV	Boundary mean CV	Inline boundary CV	Crossline boundary CV
20 × 20	2.46%	3.49%	5.33%	1.66%
10 × 10	4.05%	9.27%	15.58%	2.95%
5 × 5	9.50%	15.79%	23.39%	8.19%
3 × 3	15.45%	15.45%	25.54%	5.37%

Supplementary Table S2. Angular-bin-width sensitivity of q_{95} and the Ω_{95} M2/M1 ratio.

Field	Bin width (°)	q95 M1 (°)	q95 M2 (°)	Ω95 M2/M1
0.5 × 0.5	0.025	0.294	0.575	3.831
0.5 × 0.5	0.050	0.295	0.578	3.835
0.5 × 0.5	0.100	0.297	0.582	3.842
1 × 1	0.025	0.414	0.646	2.437
1 × 1	0.050	0.416	0.646	2.415
1 × 1	0.100	0.420	0.653	2.419
2 × 2	0.025	0.713	0.872	1.498
2 × 2	0.050	0.714	0.874	1.498
2 × 2	0.100	0.716	0.877	1.497
3 × 3	0.025	1.022	1.156	1.279
3 × 3	0.050	1.024	1.156	1.275
3 × 3	0.100	1.027	1.160	1.277

Supplementary Table S3. Directional projected q_{90} containment ratios for the prescribed M1-to-M2 endpoint change.

Field	Inline q90 M2/M1	Crossline q90 M2/M1
0.5 × 0.5	1.051 ± 0.015	1.619 ± 0.013
1 × 1	1.449 ± 0.002	1.738 ± 0.002
2 × 2	1.223 ± 0.001	1.322 ± 0.001
3 × 3	1.147 ± 0.001	1.203 ± 0.001
